# Genome-wide analysis reveals Hsf1 maintains high transcript abundance of target genes controlled by strong constitutive promoter in *Saccharomyces cerevisiae*

**DOI:** 10.1186/s13068-023-02322-2

**Published:** 2023-04-28

**Authors:** Danyao Cui, Ling Liu, Lijing Sun, Xue Lin, Liangcai Lin, Cuiying Zhang

**Affiliations:** 1 Key Laboratory of Industrial Fermentation Microbiology, Ministry of Education, Tianjin Key Laboratory of Industrial Microbiology, College of Biotechnology, Tianjin University of Science and Technology, Tianjin, 300457 People’s Republic of China; 2 State Key Laboratory of Food Nutrition and Safety, College of Biotechnology, Tianjin University of Science and Technology, Tianjin, 300457 People’s Republic of China

**Keywords:** Transcription factor Hsf1, Heat shock protein, RNA-seq, Transcription level, UPR, Ethyl acetate

## Abstract

**Background:**

In synthetic biology, the strength of promoter elements is the basis for precise regulation of target gene transcription levels, which in turn increases the yield of the target product. However, the results of many researches proved that excessive transcription levels of target genes actually reduced the yield of the target product. This phenomenon has been found in studies using different microorganisms as chassis cells, thus, it becomes a bottleneck problem to improve the yield of the target product.

**Results:**

In this study, promoters *PGK1p* and *TDH3p* with different strengths were used to regulate the transcription level of alcohol acetyl transferase encoding gene *ATF1*. The results demonstrated that the strong promoter *TDH3p* decreased the production of ethyl acetate. The results of Real-time PCR proved that the transcription level of *ATF1* decreased rapidly under the control of *TDH3p*, and the unfolded protein reaction was activated, which may be the reason for the abnormal production caused by the strong promoter. RNA-sequencing analysis showed that the overexpression of differential gene *HSP30* increased the transcriptional abundance of *ATF1* gene and production of ethyl acetate. Interestingly, deletion of the heat shock protein family (e.g., Hsp26, Hsp78, Hsp82) decreased the production of ethyl acetate, suggesting that the Hsp family was also involved in the regulation of *ATF1* gene transcription. Furthermore, the results proved that the Hsf1, an upstream transcription factor of Hsps, had a positive effect on alleviating the unfolded protein response and that overexpression of Hsf1 reprogramed the pattern of *ATF1* gene transcript levels. The combined overexpression of Hsf1 and Hsps further increased the production of ethyl acetate. In addition, kinase Rim15 may be involved in this regulatory pathway. Finally, the regulation effect of Hsf1 on recombinant strains constructed by other promoters was verified, which confirmed the universality of the strategy.

**Conclusions:**

Our results elucidated the mechanism by which Rim15–Hsf1–Hsps pathway reconstructed the repression of high transcription level stress and increased the production of target products, thereby providing new insights and application strategies for the construction of recombinant strains in synthetic biology.

**Supplementary Information:**

The online version contains supplementary material available at 10.1186/s13068-023-02322-2.

## Background

Synthetic biology is a new inter-disciplinary subject, which designs and constructs artificial biological systems based on engineering principles to solve biological problems [1]. Synthetic biology has been applied to the production of high value-added products, such as pharmaceuticals, chemicals, fragrances, amino acids and organic acids [2]. A number of strategies have been developed to increase the yield of products, such as high-efficiency transformation of products in vitro through multi-enzyme cascade reactions [3]; using biosensors to dynamically regulate gene expression in pathways to maintain metabolic balance [4]; designing artificial gene circuits to synthesize endogenous or heterologous products [5]. The fine-tuning and precise control of key gene transcription levels are the important basis for the successful implementation of these strategies.

It is beneficial to improve the protein expression and product yield by controlling the promoter strength to fine regulate the transcription level of the target gene and maximize the transcription efficiency. Several studies have demonstrated that promoter strength is positively correlated with product yield [6–8]. However, excessive promoter strength may cause metabolic flow disorder and burden on cells, which is unfavorable to the high yield of products [9, 10]. This phenomenon has been found in a number of chassis microorganisms, including *Saccharomyces cerevisiae* [11, 12], *Pichia pastoris* [13, 14], *Yarrowia lipolytica* [15, 16], *Trichoderma reesei* [17] and *Ogataea polymorpha* [18]*.* This means that the yield reduction caused by high strength promoter is a common problem and bottleneck problem in synthetic biology. One of the reasons for this phenomenon is that strong overexpression of target genes leads to protein aggregation or misfolding, and protein homeostasis is broken, resulting in cytotoxicity [11, 19, 20].

Cells have evolved a series of regulatory networks to control gene expression, precisely regulate intracellular transcriptional load, and maintain protein homeostasis under stress [21]. These protein quality control (PQC) mechanisms rely on molecular chaperones that bind to misfolded proteins, inhibit intermolecular interactions and aggregation, and then allow proteins to fold correctly and efficiently into their native forms [22, 23]. In addition to the interaction between proteins, the regulation of gene transcription level is also an important way to maintain intracellular balance. Cancer is closely related to the transcriptional levels of proto-oncogenes, which are transformed into oncogenes by high levels of overexpression, enhancing cell growth, metastasis and metabolic reprogramming, and contributing to cellular carcinogenesis [24, 25]. Therefore, exploring the effects of different transcriptional levels of genes on cellular homeostasis is important for scientific understanding of the intracellular mechanisms of transcriptional regulation.

A feedback mechanism known as repression of secretory stress (RESS) was identified in *Aspergillus niger* and *Neurospora crassa*: selective down-regulation of transcript levels of genes encoding extracellular enzymes in response to endoplasmic reticulum stress, thereby reducing endoplasmic reticulum burden [26–28]. *S. cerevisiae* has a similar functional mechanism, the unfolded protein response (UPR), but the mechanism of UPR regulation of intracellular transcription levels and the relationship with other pathways need to be further explored. In this study, *S. cerevisiae* was used as host strain to simulate the repression of high transcription level stress (RHTS) using promoters with different strengths. Our results demonstrated that the expression of transcription factor Hsf1 and molecular chaperones Hsp26, Hsp42, Hsp78, and Hsp82 alleviated the repression of *ATF1* gene transcription level and significantly increased the yield of ethyl acetate. In this study, the negative effects of target gene overexpression on cells homeostasis were clarified by controlling the promoter strength, and a new strategy to alleviate RHTS was elaborated. It provides a new idea for the production of high value-added natural products using synthetic biology and metabolic engineering.

## Methods

### Media and cultivation

*Saccharomyces cerevisiae* was grown in YPD medium at 30 °C consisting of 2% glucose, 1% yeast extract and 2% peptone. The recombinant strain was screened using a YPD plate containing 300 μg/mL G418 (Promega, Madison, U.S.A.). The preparation of yeast extract peptone galactose medium was as follows: 1% yeast extract, 2% peptone, 2% galactose. All solid media ere added with 2% agar powder (Solarbio, Beijing, China).

### Construction of mutant strains

Strain lists were shown in Additional file 1: Table S1. In this study, the strategy of homologous recombination was utilized to construct mutant strains. The recombinant cassette was transformed into *S. cerevisiae* by lithium acetate/polyethylene glycol (PEG) and verified by polymerase chain reaction (PCR). The *KanMX* gene was removed by *Cre/loxP* system [29]. Primers were listed in Additional file 1: Table S2.

### Fermentation experiments

Two fermentation media were used to test the fermentation performance of the parental or construction strains, namely, YPD-20 medium and corn synthetic medium. The YPD-20 medium contained 20% glucose, 5% peptone and 2% yeast extract. Yeast cells were inoculated in 15 mL of YPD medium (distributed in 50 mL centrifuge tubes), and cultured with shaking at 180 rpm for 12 h. After the OD_600_ value was adjusted to the same level, 2 mL of yeast suspension was inoculated into 98 mL of YPD-20 medium (distributed in 250 mL conical flasks), and cultured at 30 °C by standing or shaking. The preparation method and inoculation strategy of corn synthesis medium was referred to the study of Cui et al. [30]. After the fermentation, 100 mL of the fermentation broth was mixed with 100 mL of water, and the samples to be tested were obtained after distillation.

### Detection of ethyl acetate and related metabolites

The productions of ethyl acetate and related metabolites were quantified by gas chromatography (GC) [31]. Agilent 7890C GC was equipped with the HPINNOWax polyethylene glycol (30 m × 320 μm i.d. and a 0.5 μm coating thickness, organic-coated fused silica capillary column). An internal calibration curve was constructed using standard compounds of specific concentrations to quantify the compounds.

### Fluorescence strength detection

Red fluorescent protein (RFP) and enhanced green fluorescent protein (EGFP) were used as reporter genes to characterize promoter strength. The strains were grown in YPD medium for 12 h, 1 mL of the culture was extracted, and cells were obtained by centrifugation. Cells suspended twice in phosphate-buffered saline buffer were assayed for fluorescence strength in a Synergy 4 Multi-Detection microplate reader (BioTek, Winooski, VT). The fluorescence strength detection conditions of EGFP and RFP were different: the excitation and emission wavelengths of EGFP are 398 nm and 510 nm, while the excitation and emission wavelengths of RFP are 585 nm and 625 nm. Meanwhile, the OD_600_ of the cells was detected and the relative fluorescence strength was calculated by comparing the fluorescence strength with the OD_600_ value.

### Real-time quantitative PCR

Total RNA extraction and cDNA synthesis for RT-PCR were performed by Yeast Processing Reagent (Takara Biotechnol, Dalian, China) and PrimeScript™ RT reagent Kit (Takara Biotechnol, Dalian, China), respectively. The transcript levels of *ATF1*, *HSP26*, *POR1*, *GRE1*, *HPA3*, *TIR1*, *PUT4*, *HSP30*, *DIP5*, *ALD2*, *YER188W*, *NIS1*, *ALD6*, *ADH1*, *ADH2*, *MET10*, *HSP42*, *HSP78* gene in the strains were detected by quantitative real-time PCR (RT-qPCR) with TB Green® Premix EX Taq™ II (Tli RNaseH Plus; Takara Biotechnol, Dalian, China). The *UBC6* gene was the reference gene and the 2^−ΔΔCt^ method was used for quantitative analysis of gene transcript levels. All Primers used for RT-PCR were listed in Additional file 1: Table S2.

### RNA sequencing and data analysis

For RNA-seq experiments, strains IS45, ISP, ISH were fermented in corn synthetic medium, and statically cultured for 72 h at 30 °C. Yeast cells in the fermentation broth were collected by cryogenic centrifugation at the timepoint (12, 24, 36 h) and immediately frozen in liquid nitrogen. After extraction of total RNA, Oligo(dT) beads were used to enrich mRNA. And then, the enriched mRNA was fragmented into short fragments, and reversely transcribed into cDNA with random primers. Second-strand cDNA was synthesized by DNA polymerase I, RNase H, dNTP and buffer. The cDNA fragments were purified and end repaired, poly(A) added, and ligated to Illumina sequencing adapters. The ligation products were size selected by agarose gel electrophoresis, PCR amplified, and sequenced using Illumina HiSeqTM4000 by Gene Denovo Biotechnology Co. (Guangzhou, China).

The RNA-seq data have been submitted to NCBI (accession ID: PRJNA899334). RNA-seq reads were aligned to reference strain *Saccharomyces cerevisiae* S288c (Accession: GCF_000146045.2_R64) genome. Differential expression analysis was performed with DESeq2, transcripts with *P* value < 0.05 and the value of log_2_(Foldchange) ≥ 1 were considered as differentially expressed genes (DEGs). The topGO and cluster Profiler were used for enrichment analysis of Gene Ontology (GO) and Kyoto Encyclopedia of Genes and Genomes (KEGG), respectively. The GO or KEGG terms in DEGs were considered as significantly enriched when their corrected *P* value < 0.05.

### Determination of the growth curve

First, the yeast strains were incubated at 180 rpm for 12 h in a test tube containing 5 mL YPD medium. Then, the amounts of yeast cells were adjusted and transferred to a 96-well plate containing fresh YPD medium. Finally, the optical density (OD_600_) was measured every 0.5 h for 12 h using Bioscreen Automated Growth Curves analysis system (OY Growth Curves Ab Ltd., Helsinki, Finland).

### Statistical analysis

Data were represented as mean ± standard error. Student’s *t* test was used for the calculation of the differences between the transformants and the parental strain. The *P* value < 0.05 was considered statistically significant.

## Results

### The promoter with high strength is unfavorable to high yield of product

**Fig. 1:**
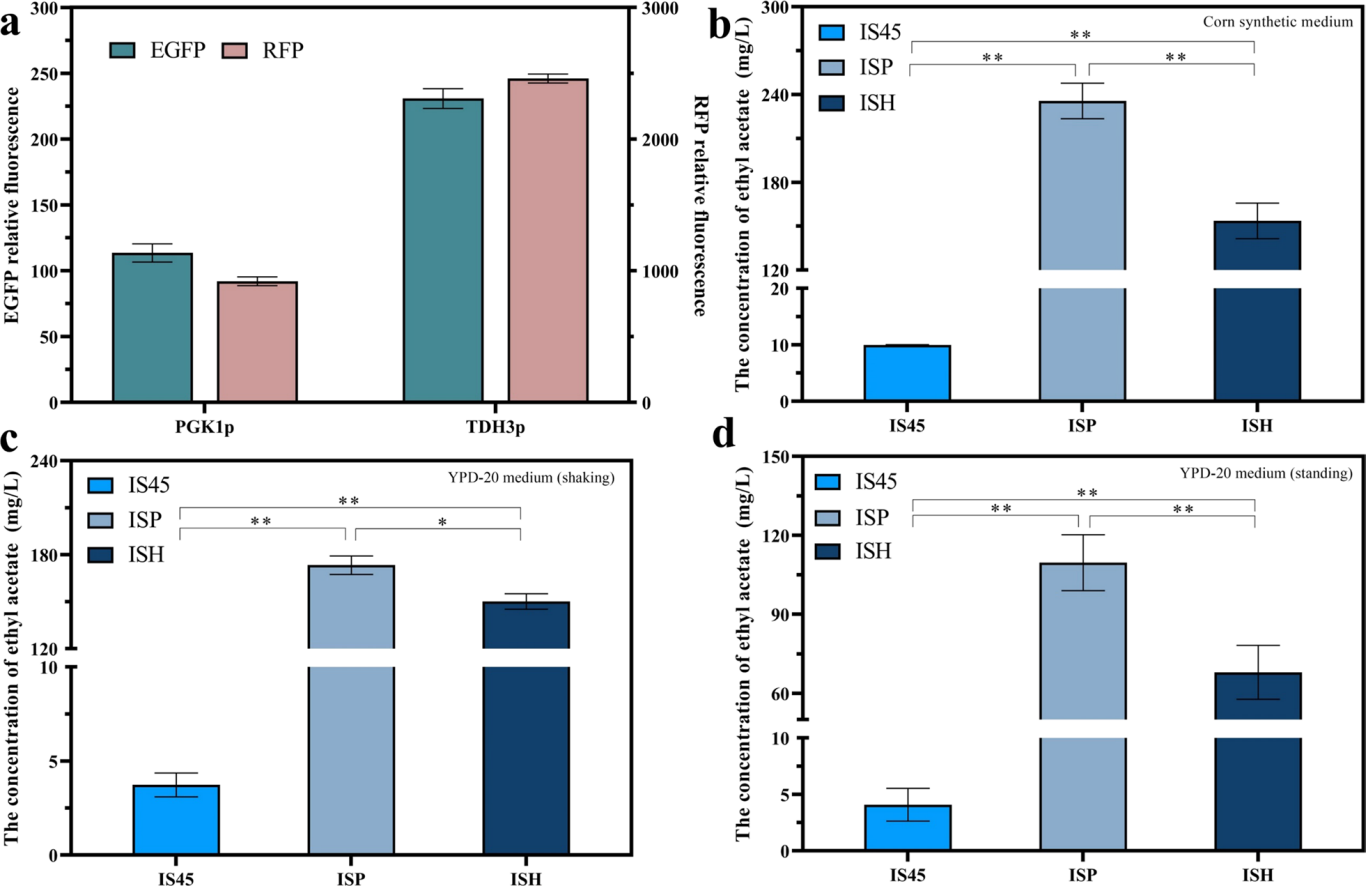
Effect of overexpression of *ATF1* by different promoters on the regulation of ethyl acetate production. **a** Identification of promoter strength of *PGK1p* and *TDH3p*. Metabolite productions of strains IS45, ISP, ISH in corn synthesis medium for standing fermentation (**b**), YPD-20 medium for shaking (**c**) and standing fermentation (**d**). The value represented mean ± SD (*n* = 3)

Ethyl acetate is an important flavors, green solvents and advanced biofuels [32–34]. In *S. cerevisiae*, alcohol acetyl transferase encoded by *ATF1* gene is a key enzyme in the synthesis of ethyl acetate. In our previous experiments, a *PGK1p* mutant library was constructed by error-prone PCR method, and the *PGK1p* mutant in the library was used to regulate the expression of *ATF1* gene, and recombinant strains with different ethyl acetate yields were obtained [31]. However, the results showed that the mutant strain constructed with increased strength of *PGK1p* (compared with wild-type *PGK1p*) had lower ethyl acetate production than the wild-type strain instead. Therefore, to elucidate this phenomenon, the constitutive promoter *TDH3p* and *PGK1p* were selected to overexpress the *ATF1* gene. These promoters are *PGK1p*, the promoter of phosphoglycerate kinase, and *TDH3p,* the promoter of glyceraldehyde-3-phosphate dehydrogenase, which had extremely broad applications in metabolic engineering and synthetic biology [35]. Among them, *TDH3p* was even considered to be the highest strength constitutive promoter in *S. cerevisiae* [36]. Red fluorescent protein (RFP) and enhanced green fluorescent protein (EGFP) were used as reporter genes, and the promoter strength of *PGK1p* and *TDH3p* were determined. In Fig. 1a, the relative fluorescence strength of *TDH3p* was 2.03-folds higher than that of *PGK1p* when EGFP was used as a reporter gene. The same result was obtained with RFP as a reporter gene, which indicated that the strength of *TDH3p* was higher than that of *PGK1p*.

The mutant strains ISP and ISH overexpressing *ATF1* gene were constructed using industrial strain IS45 as the parental strain, and *PGK1p* and *TDH3p* as promoters, respectively. Due to the strength of *TDH3p* was higher than that of *PGK1p*, we conjectured that the effect of strain ISH on the improvement of ethyl acetate production may be lower than that of ISP. To analysis the ability of strains IS45, ISP and ISH to produce ethyl acetate, strains were fermented in corn synthetic medium. As shown in Fig. 1b, the ethyl acetate production of strain ISP and ISH fermented in corn synthetic medium was higher than that of parental strain IS45. Moreover, the ethyl acetate production of ISP was 1.53 times that of strain ISH. To investigate the effect of different culture conditions on ethyl acetate production, the strain was fermented in YPD medium (YPD-20) containing 20% glucose. As shown in Fig. 1c, d, the ethyl acetate production of strain ISP was higher than that of strain ISH, whether in shaking culture or stationary culture. As shown in Additional file 1: Fig. S1 and Table S3, there were no differences in growth curves, ethanol production, reducing sugar consumption and CO_2_ release among the three strains, and no changes in growth and fermentation performance. This suggests that the abnormal decrease in ethyl acetate production was not caused by the fermentation performance and growth defects of the strain. This confirmed our conjecture that ethyl acetate production was inferior to that of the strains constructed with less intense promoters when the higher strength promoter overexpressed the *ATF1* gene.

### Comparative analysis of transcriptomes between ISH, ISP and IS45 strains

**Fig. 2:**
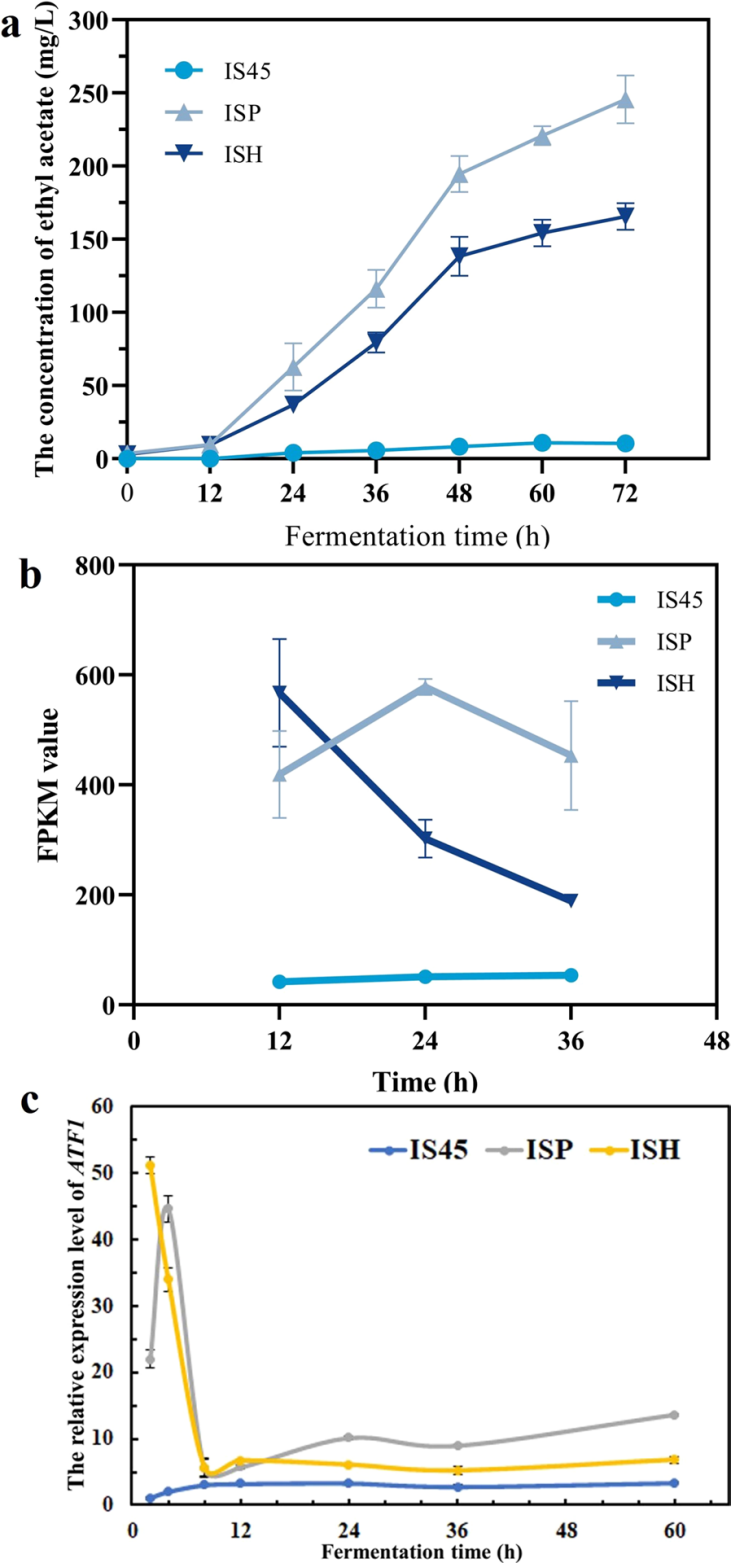
Effect of *ATF1* gene overexpression on global transcript levels. **a** Ethyl acetate production of strains IS45, ISP and ISH at different timepoints. The value represented mean ± SD (*n* = 3). **b** Changes in FPKM values of *ATF1* gene in strains IS45, ISP and ISH. The value represented mean ± SD (*n* = 2). **c** Validation of transcript levels of *ATF1* gene by RT-PCR. The value represented mean ± SD (*n* = 3)

To determine the extent of the transcriptional changes conferred by overexpression of the *ATF1* gene at different strength promoters, we determined dynamitic changes in the expression of genomic genes during the fermentation by transcriptome sequencing. The ethyl acetate production of three strains were detected every 12 h during the fermentation process. As shown in Fig. 2a, the trend in the content of ethyl acetate during the 72-h fermentation period was gradually increasing without fluctuations. Therefore, at 12, 24 and 36 h of the fermentation process, the yeast cells in the fermentation broth were collected and then subjected to transcriptome sequencing.

The changes of FPKM value of *ATF1* gene during the fermentation were presented in Fig. 2b. The transcription level of *ATF1* gene of ISH strain gradually decreased at 12, 24 and 36 h, and were significantly lower than ISP after 24 h. However, the expression level of *ATF1* gene was more stable in ISP compared to strain ISH. This result explains the decrease in ethyl acetate production but is not consistent with the detection of promoter strength. To obtain more information about the changes in *ATF1* gene transcript levels with fermentation time, samples from the early (2 h, 4 h, 8 h), middle (12 h, 24 h, 36 h) and late (60 h) stages of fermentation were examined by RT-PCR. As shown in Fig. 2c, at the beginning of fermentation (2 h), strain ISH had the highest level of *ATF1* gene transcription, 51.17-fold higher than that of parental strain IS45, followed by strain ISP (21.96-fold). This was consistent with the results of the promoter strength detection, which indicated that the strength of the promoter at the beginning of fermentation determined the transcriptional strength of the target gene *ATF1*. However, after the 4th hour of fermentation, the *ATF1* gene expression level of strain ISH continued to decrease until 8 h and then fluctuated to stabilize. However, the *ATF1* expression level of ISP strain showed a totally different expression pattern. The transcriptional level was sharply increased to 44.62-fold (compared to IS45) at 8 h, and then dramatic dropped during the next 4 h, finally its level was stable but lower than that of ISP at the latter period. At 12–60 h, the *ATF1* gene expression level tended to be stable without dramatic fluctuations. The *ATF1* expression level of the ISP strain was higher than that of ISH, and more importantly, the change trend was consistent with the transcriptome sequencing results. These results demonstrated that the transcriptional level of *ATF1* was repressed during fermentation, and the higher the promoter strength, the more pronounced its repressive effect. This may be one of the reasons for the abnormal production of ethyl acetate.

**Fig. 3:**
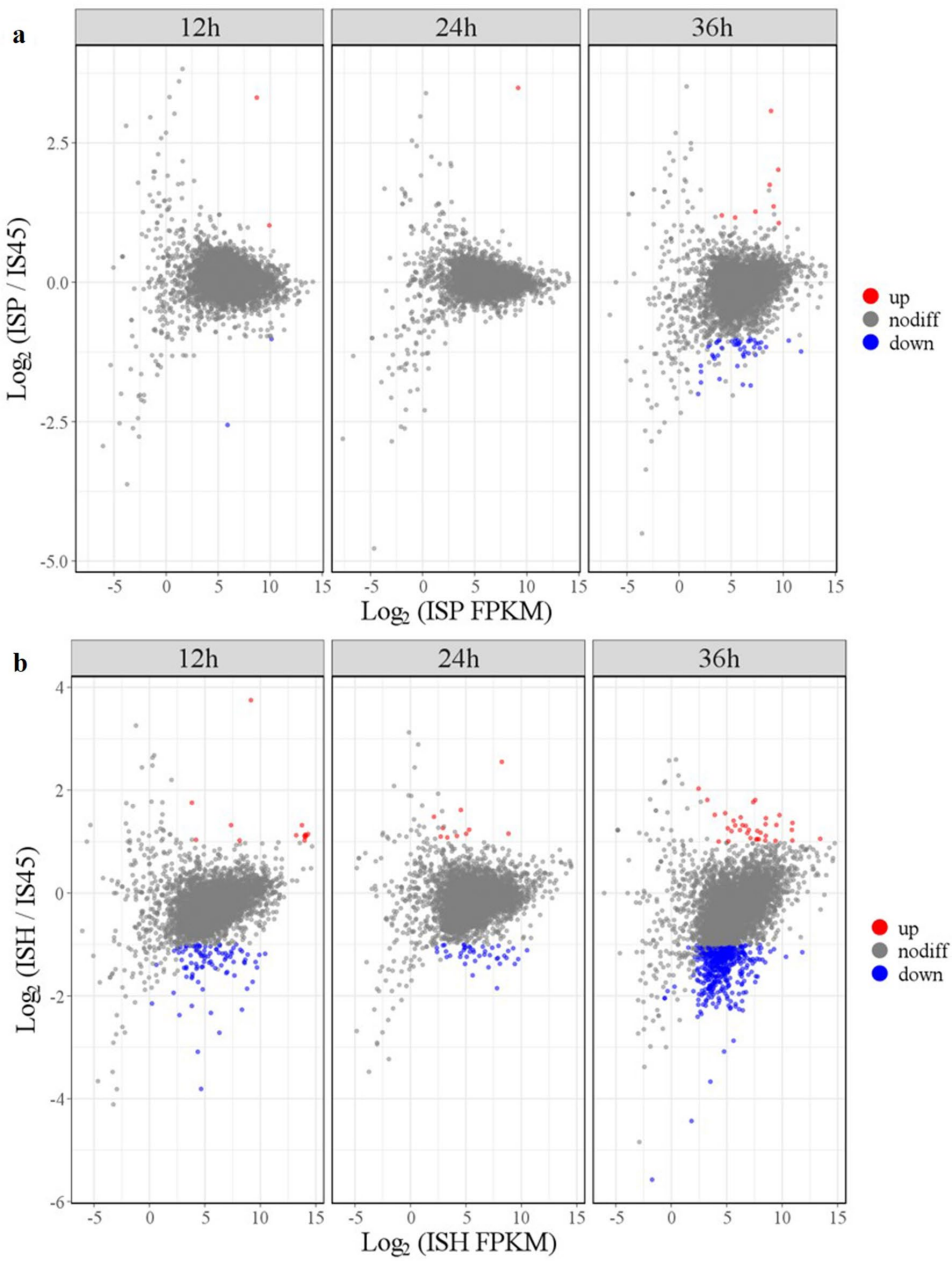
Global gene transcript level profiling of strains ISP and ISH. Red points and blue points represent up-regulation and down-regulation of DEGs transcript levels in strains ISP (**a**) and ISH (**b**), respectively. Gray points represent genes with no differences in transcript levels between IS45 and ISP/ISH

As shown in Fig. 3, The number of DEGs gradually increased with fermentation time, at the 12th hour, compared with the strain IS45, the number of up-regulated DEGs in mutant strains ISP and ISH were 9 and 21, respectively; the down-regulated DEGs were 7 and 103, respectively. The number of DEGs increased with fermentation time: at 36th hour, the number of up-regulated DEGs reached 12 and 36 for strains ISP and ISH; the number of down-regulated genes was 63 and 594. GO enrichment analysis of DEGs in three strains at 12, 24, 36 h was completed, and the results were shown in Additional file 1: Fig. S2. For molecular function ontology, overexpression of the *ATF1* gene affected the expression of genes mainly involved in catalytic activity, binding, transporter activity, nucleic acid binding transcription factor activity in mutant strains. For cellular component ontology, the genes mainly involved in cell, cell part, organelle, membrane, macromolecular complex differentially expressed after stress. In addition, for biological process, the DEGs were mainly categorized into single-organism process, cellular process, metabolic process, localization, cellular component organization or biogenesis.

KEGG pathway enrichment analysis was also applied to the analysis of DEGs. The results of DEGs enrichment in the KEGG pathway between the mutant strains ISH and ISP and the parental strain IS45 were shown in Additional file 1: Fig. S3. Some common pathways were significantly enriched in the comparison groups at different times, including Metabolic pathways (ID: ko01100), Biosynthesis of secondary metabolites (ID: ko01110), Biosynthesis of antibiotics (ID: ko01130), Carbon metabolism (ID: ko01200), Biosynthesis of amino acids (ID: ko01230). This indicated that these pathways were deeply associated with the decrease in ethyl acetate production caused by the strong promoter.

Moreover, RT-PCR was used to verify the reliability of transcriptome data. Cells of strain ISP and ISH were isolated from the 36 h fermentation broth as samples, and transcript levels of 17 genes (*HSP26*, *POR1*, *GRE1*, *HPA3*, *TIR1*, *PUT4*, *HSP30*, *DIP5*, *ALD2*, *YER188W*, *NIS1*, *ALD6*, *ADH1*, *ADH2*, *MET10*, *HSP42*, *HSP78*) were examined and then compared with data from the transcriptome. As shown in Additional file 1: Fig. S4, the correlation coefficients (*R*^2^ value) of the trend lines for strain ISP and ISH were 0.918 and 0.9205, respectively. These validation results show that the transcriptome sequencing data are reliable and provide support for the in-depth study of RHTS phenomenon caused by high-intensity promoters.

### Effect of DEGs on RHTS

DEGs were screened from RNA-seq data to analyze their effect on RHTS. Based on the repression of *ATF1* gene transcript levels in strain ISH during fermentation, we hypothesized that repression of high transcription level stress was activated. Therefore, the selected target DEGs were mainly kinases, transcription factors and stress-related genes with more than fourfold change in FPKM value. Ultimately, the expression levels of adenylate sulfate kinase Met14, aspartate kinase Hom3, DNA-binding transcription factor Adr1, transcription factor Hap4, protein Btn2 and heat shock protein Hsp30 of strain ISH were differentially expressed compared to strain IS45 and were considered as DEGs. The comparison of FPKM values of these DEGs at 12, 24, 36 h was presented in Additional file 1: Fig. S5. The FPKM values of *MET14*, *HOM3*, *ADR1*, *HAP4*, *HSP30*, and *BTN2* genes were decreased by 76.71%, 78.64%, 76.46%, 75.92%, 79.45%, and 92.13% in the ISH-36 sample compared with the IS45-36 sample, respectively.

**Fig. 4:**
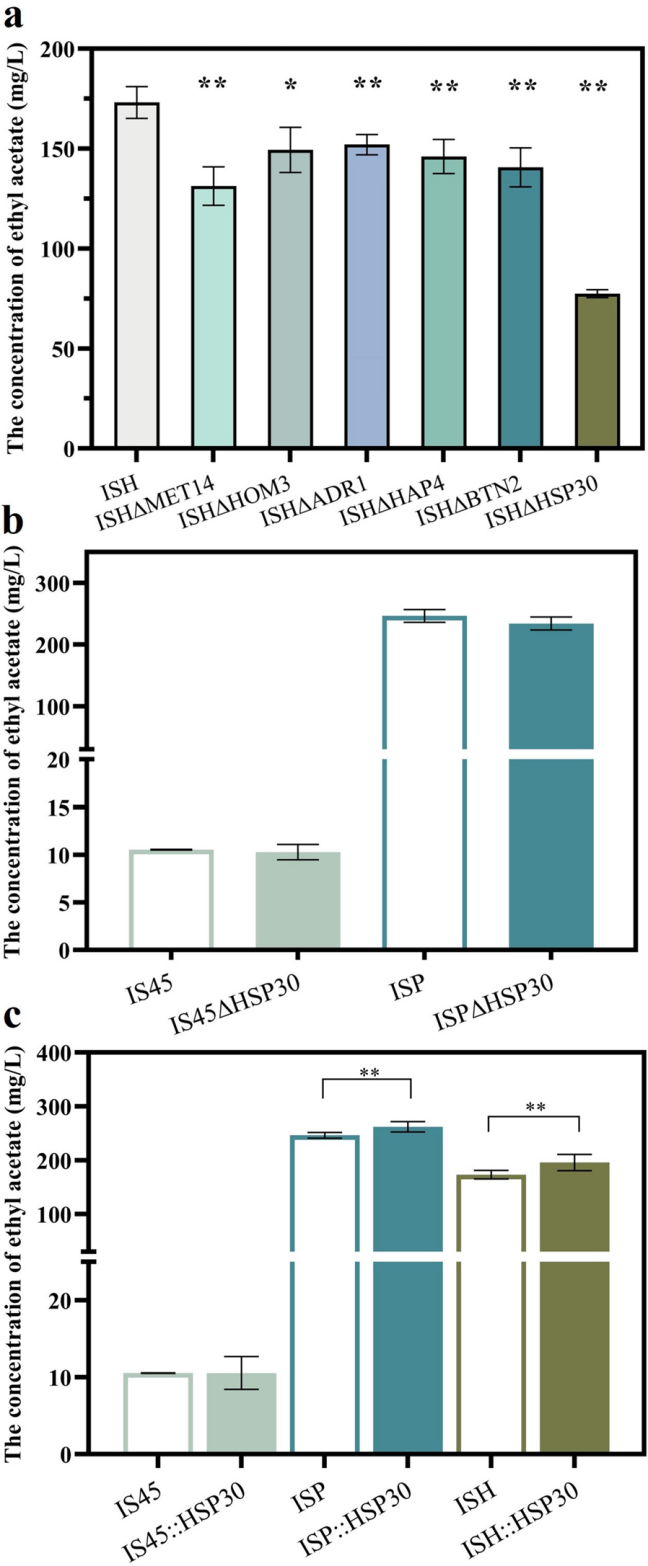
Effect of the DEGs deletion on the concentration of ethyl acetate production. **a** Ethyl acetate production of *MET14*, *HOM3*, *ADR1*, *HAP4*, *HSP30*, *BTN2* gene deletion strains. **b** Ethyl acetate production of strains IS45∆HSP30 and ISP∆HSP30. **c** The concentration of acetate ethyl produced by *HSP30* gene overexpression strains. These recombinant strains were fermented in corn synthesis medium. The value represented mean ± SD (*n* = 3). Statistical significance is denoted as **P < 0.01, *P < 0.05

The corresponding *MET14*, *HOM3*, *ADR1*, *HAP4*, *BTN2* and *HSP30* gene deletion mutant strains were constructed by deletion strategy using ISH as the parental strains to examine their effects on ethyl acetate production. As shown in Fig. 4a, the deletion of six DEGs resulted in the decrease of ethyl acetate production, but the extent of the decrease was different. The ethyl acetate production of strain ISH∆MET14, ISH∆HOM3, ISH∆ADR1, ISH∆HAP4, ISH∆BTN2 and ISH∆HSP30 was 131.31, 149.33, 152.06, 146.11, 140.66 and 77.46 mg/L, which decreased by 24.17%, 13.75%, 12.18%, 15.61%, 18.76% and 55.26% compared with ISH strain, respectively. It was obvious that the deletion of *HSP30* gene interfered most obviously with the production of ethyl acetate. Then, strains IS45∆HSP30 and ISP∆HSP30 were constructed to verify the down-regulation effect of knocking out *HSP30* in different strains on ethyl acetate production (Fig. 4b). The ethyl acetate production of strains IS45∆HSP30 and ISP∆HSP30 was similar to that of their respective parental strains, implying that the *HSP30* gene functioned only at high transcriptional stress.

It was well-known that the *HSP30* gene encodes the plasma membrane heat shock protein involved in responses to heat stress, ethanol stress, DNA damage, and negative regulation of ATPase activity [37]. It has been described that *HSP30* gene has a mitigating function for protein misfolding caused by stress response. Therefore, we overexpressed the *HSP30* gene in strains IS45, ISP and ISH to verify whether they had a positive alleviating effect on the RHTS of the strains. As shown in Fig. 4c, the ethyl acetate production of strain ISH::HSP30 was 13.11% higher than that of strain ISH. The ethyl acetate production of strain ISP::HSP30 also showed an increasing trend. This indicated that the overexpression of *HSP30* gene had an alleviating effect on the repression of target gene transcription level caused by high promoter strength. Furthermore, the expression of genes related to ethyl acetate metabolism was also analyzed, aldehyde dehydrogenase coding genes *ALD2*, *ALD6* and alcohol dehydrogenase coding genes *ADH1*, *ADH2* genes were considered as DEGs. However, deletion of these genes did not affect the production of ethyl acetate (Additional file 1: Fig. S6). Therefore, it indicated that the decrease of ethyl acetate production in strain ISH was not caused by metabolic network regulation. The repression of *ATF1* gene transcription level was the most important potential reason for the decrease in ethyl acetate production.

### Alleviating effect of heat shock protein family on RHTS

**Fig. 5:**
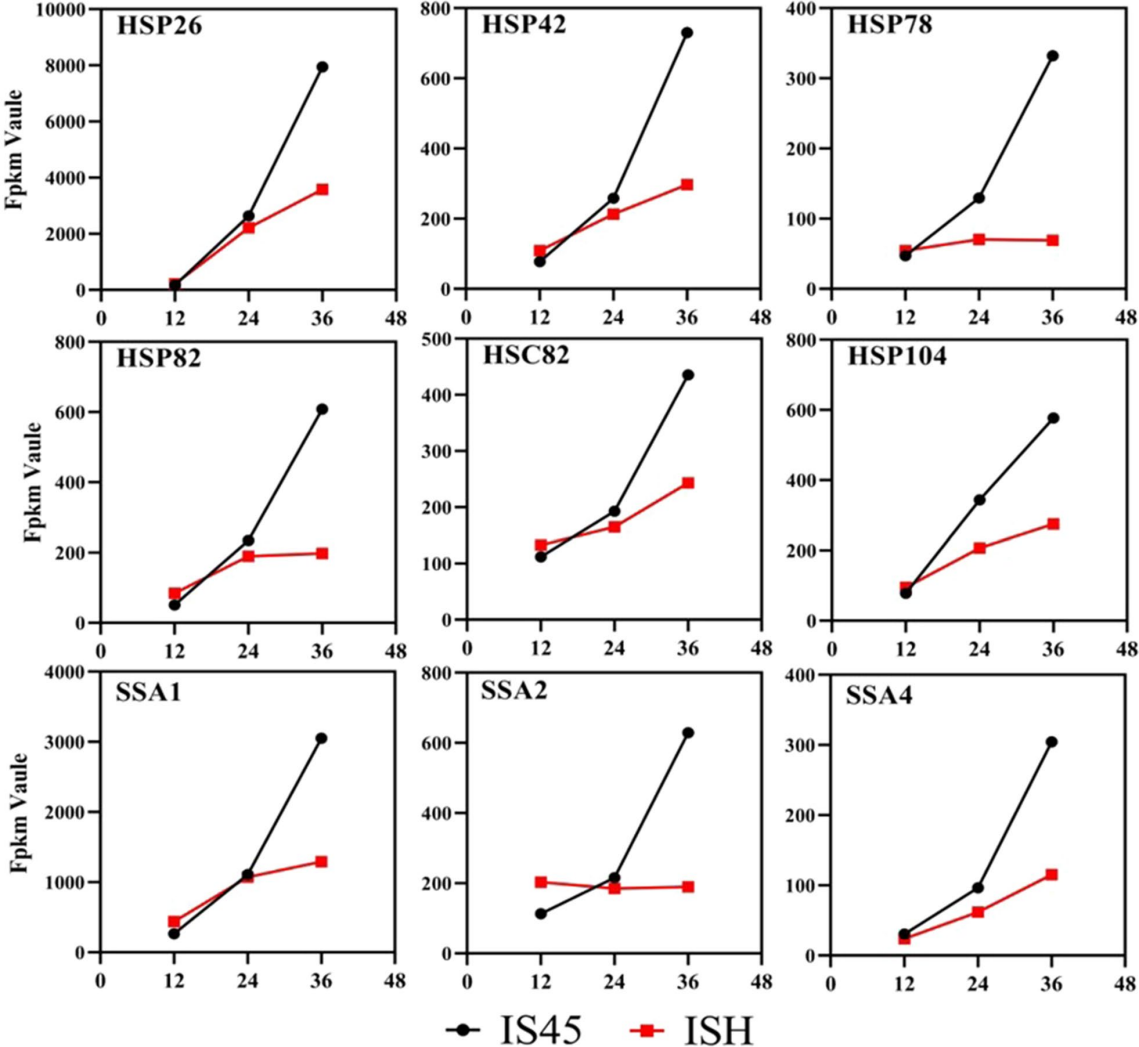
Schematic diagram of transcription levels (FPKM values) of some heat shock protein coding genes at different times. The horizontal coordinate represents the fermentation time and the vertical coordinate represents the FPKM value. The change curves of FPKM values for strains IS45 and ISH are shown in black and red, respectively. Gene names are labeled in the upper left corner of each sub-figure

As mentioned above, overexpression of *HSP30* gene alleviated the repression caused by high transcript levels of target genes, implying that the Hsp family may be involved in RHTS. Therefore, other elements of the Hsp family were examined in response to high transcription. Based on molecular weight, heat shock proteins are divided into five main families, including: Hsp100, Hsp90, Hsp70, Hsp60 and small Hsp [38]. The FPKM values of heat shock protein-related genes in strain IS45 and ISH were analyzed and compared. As shown in Fig. 5, the transcript levels of *HSP26*, *HSP42*, *HSP78*, *HSP82*, *HSC82*, *HSP104*, *SSA1*, *SSA2* and *SSA4* genes in strain ISH were all lower than those in wild-type strain IS45, suggesting that they might also be involved in cell responses induced by high transcript abundance of *ATF1* gene.

**Fig. 6:**
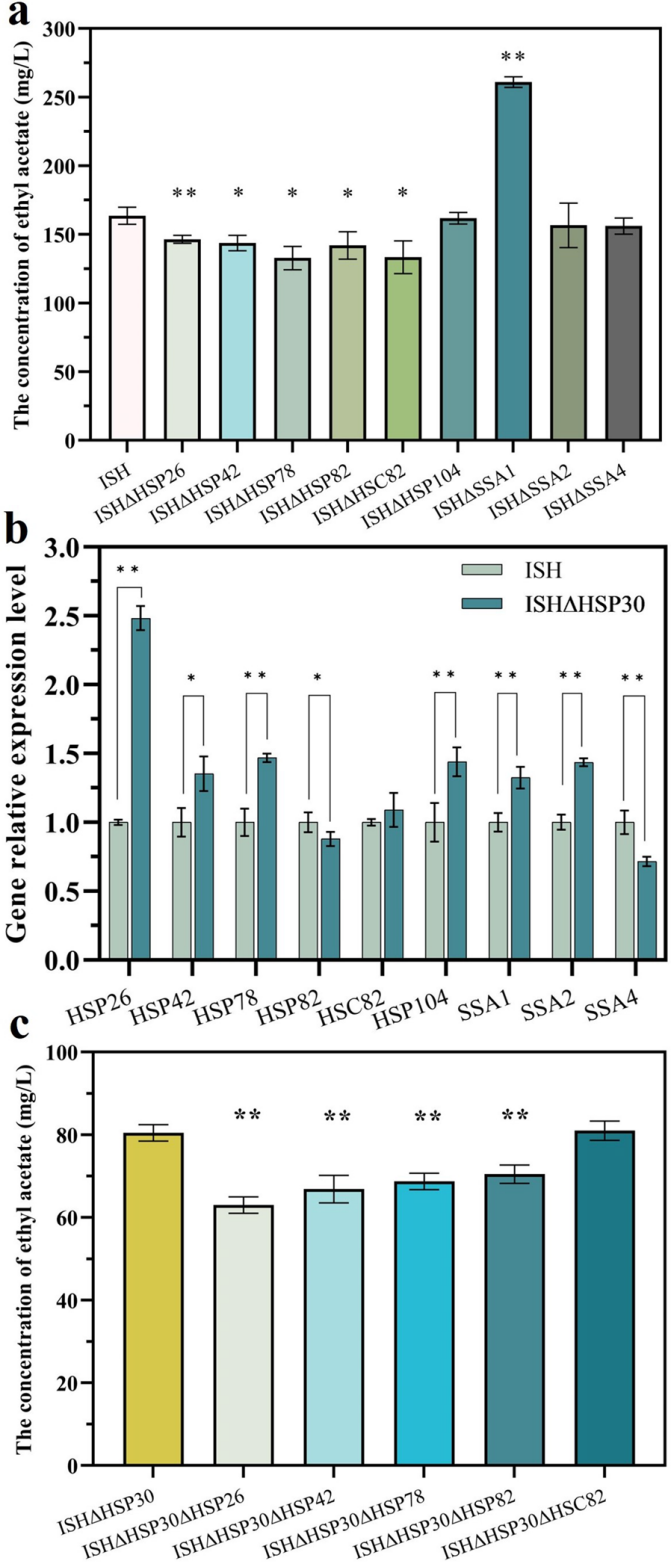
Regulatory effects of the heat shock protein family on RHTS. **a** Effect of deleting *HSP26*, *HSP42*, *HSP78*, *HSP82*, *HSC82*, *HSP104*, *SSA1*, *SSA2* and *SSA4* genes on ethyl acetate production using ISH as parental strain. **b** Comparison of the transcript levels of *HSP26*, *HSP42*, *HSP78*, *HSP82*, *HSC82*, *HSP104*, *SSA1*, *SSA2*, *SSA4* genes in strains ISH and ISH∆HSP30. **c** Effect of deleting *HSP26*, *HSP42*, *HSP78*, *HSP82*, *HSC82* genes on ethyl acetate production using ISH∆HSP30 as parental strain. The value represented mean ± SD (*n* = 3). Statistical significance is denoted as **P < 0.01, *P < 0.05

To investigate the alleviating effect of these nine genes on RHTS, the knockout strategy was applied to the mutant strain ISH. As shown in Fig. 6a, the content of ethyl acetate produced by strains deleted *HSP26*, *HSP42*, *HSP78*, *HSP82* and *HSC82* genes was 146.48, 143.77, 132.86, 142 and 133.86 mg/L, respectively, which was reduced by 10.49%, 12.14%, 18.81%, 13.23% and 18.44%, respectively, compared with strain ISH, suggesting that increasing the expression of these genes might alleviate the repression induced by high transcript abundance of *ATF1* gene. Intriguingly, *SSA1* might have an opposite function. Our results showed that the ethyl acetate production of ISH∆SSA1 with *SSA1* gene knockout was 59.44% higher than that of its parental strain. Furthermore, there was no significant difference in the content of ethyl acetate produced by strain ISH∆HSP104, ISH∆SSA2, ISH∆SSA4 compared to strain ISH. The growth performance was not affected by single deletion of heat shock protein encoding genes (shown in Additional file 1: Fig. S7). These results may imply that Hsp26, Hsp42, Hsp78, Hsp82 and Hsc82 proteins had similar functions to Hsp30 under the condition of high transcription levels of target genes.

The transcript levels of the *HSP26*, *HSP42*, *HSP78*, *HSP82*, *HSC82*, *HSP104*, *SSA1*, *SSA2* and *SSA4* genes in strain ISH∆HSP30 were detected to analyze the correlation between Hsp30 and other Hsps. As shown in Fig. 6b, deletion of the *HSP30* gene resulted in a slight upregulation of the transcript levels of most of the Hsps (except for Hsp82 and Ssa4). Among them, the transcript level of *HSP26* gene was most significantly up-regulated by 2.48-fold. This suggested that the transcript levels of *HSP30* and *HSP26* were complementary and may work together to maintain the functional integrity of Hsp family.

To further explore the role of *HSP30* gene and Hsp family in RHTS, *HSP26*, *HSP42*, *HSP78*, *HSP82* and *HSC82* genes were further deleted on the basis of *HSP30* gene knockout. As shown in Fig. 6c, due to the complementary functions of Hsp26 and Hsp30, their combined knockout further affected the intracellular RHTS. Therefore, the ethyl acetate production of strain ISHΔHSP30ΔHSP26 was most significantly reduced, which was 21.72% lower than that of strain ISHΔHSP30. In addition, the ethyl acetate concentration of the mutant ISHΔHSP30ΔHSP42, ISHΔHSP30ΔHSP78 and ISHΔHSP30ΔHSP82 decreased by 16.89%, 14.57% and 12.4%, respectively. These results indicated that Hsp26, Hsp42, Hsp78 and Hsp82 played an important role in maintaining the production of target products in response to strong promotor-induced stress. Transcription levels of these heat shock protein-coding genes were down-regulated in strain ISH, which may be one of the reasons for the abnormal decrease of ethyl acetate production in strain ISH.

### The high transcript levels of target genes induced UPR

According to the previous investigations, heat shock proteins are important molecular chaperones for maintaining protein homeostasis in *S. cerevisiae*. Increasing the expression level of heat shock proteins is beneficial for maintaining the correct protein conformation of yeast cells under heat stress. In addition to the heat shock response (HSR), the unfolded protein response (UPR) is also associated with the cellular response to stress and the repair of protein function [39–41]. These results implied that UPR might be associated with RHTS, and to confirm this speculation, the transcript levels of UPR signature elements were examined. When unfolded proteins aggregate in the endoplasmic reticulum (ER), the endoribonuclease Ire1 is activated to unconventionally splice *HAC1* mRNA, forming the active transcription factor Hac1. Hac1 enters the nucleus to bind to UPR sequences on the promoters of downstream target genes, relieving ER stress. We have demonstrated that heat shock proteins show a positive effect on relieving transcriptional stress, and thus speculate whether there is also a correlation between the stress response induced by high transcription levels and UPR.

**Fig. 7:**
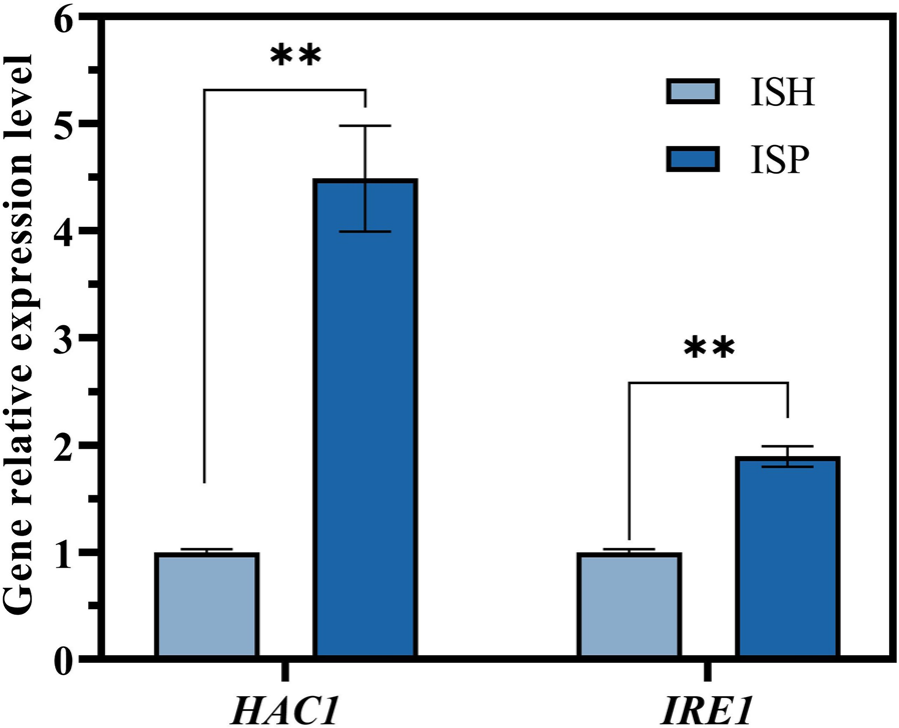
Detection of relative transcription levels of *HAC1* and *IRE1* genes. Relative transcription levels of *HAC1* and *IRE1* genes in strain ISH compared with that of strain ISP. The value represented mean ± SD (*n* = 3). Statistical significance is denoted as **P < 0.01, *P < 0.05

Thus, as signature genes in the UPR pathway, *IRE1* and *HAC1* transcript levels were detected in strains ISH and ISP. As shown in the Fig. 7, the transcript level of *HAC1* gene and *IRE1* gene was increased 4.48-fold and 1.90-fold in ISH, respectively, using strain ISP as a control. This indicated that the UPR in ISH strain was activated more obviously. We hypothesized that under stress conditions caused by high transcription levels, the aggregation of unfolded proteins in cells is more severe, activating UPR. The RHTS mediated by heat shock protein is associated with the UPR pathway.

### Overexpression of transcription factor Hsf1 alleviated the negative effect of RHTS on production

According to the above experimental results, HSR and UPR pathways have been confirmed to be involved in ethyl acetate synthesis under stress regulation of high transcription levels. Although the overexpression of *HSP30* increased the ethyl acetate production of ISH strain, it was still lower than that of ISP strain. Therefore, we try to regulate transcription factors to decrease the stress response of cells. It has been known that the expression of *HSP30* gene is regulated by transcription factors Hsf1, Msn2 and Msn4 simultaneously [42]. Therefore, three transcription factors were overexpressed in strains ISH and ISH::HSP30 to verify their regulatory effect on ethyl acetate production and the functional association between transcription factors and *HSP30* gene.

**Fig. 8:**
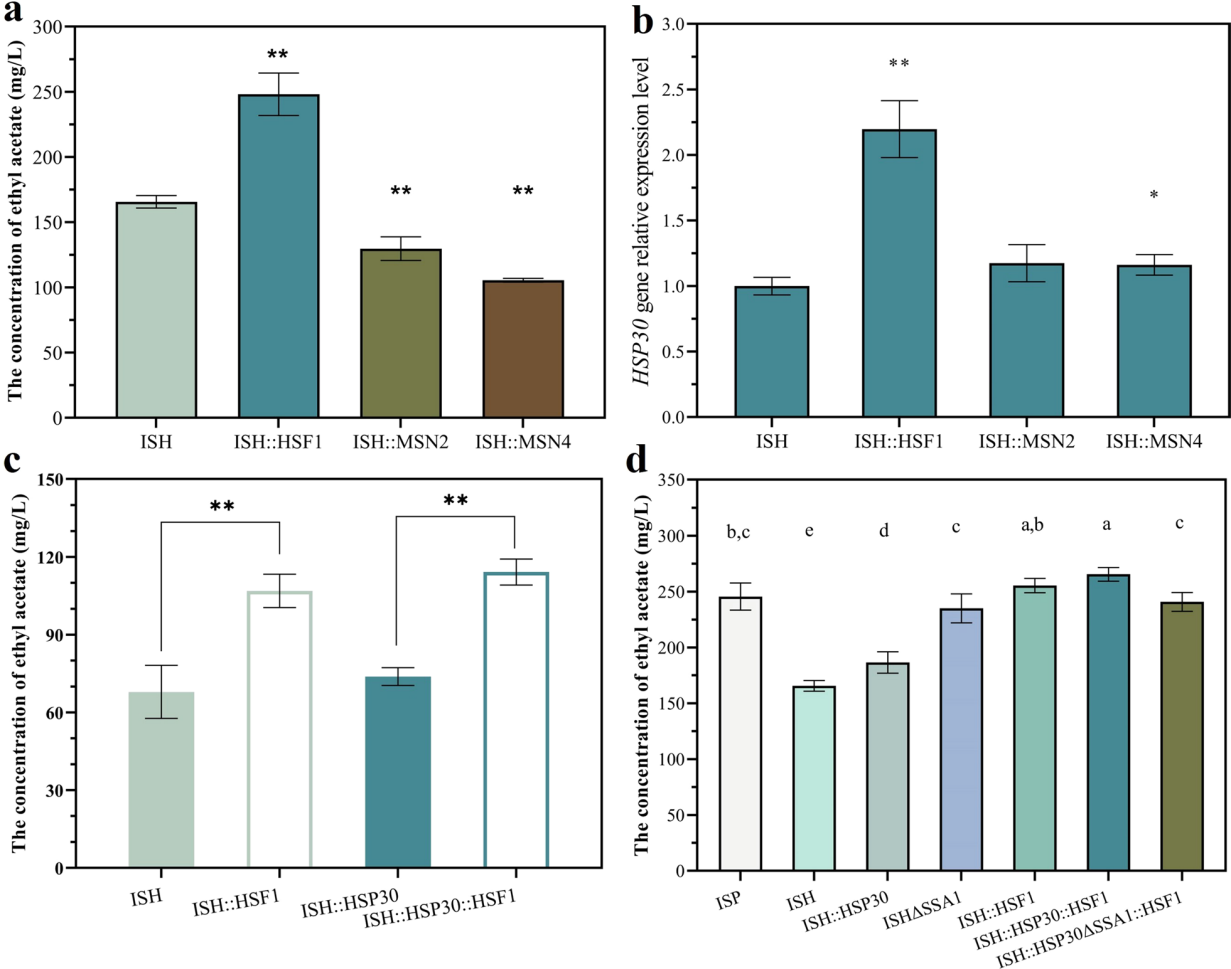
Effect of transcription factors Hsf1, Msn2 and Msn4 on the concentration of ethyl acetate production. **a** Ethyl acetate production of mutant strains overexpressing *HSF1*, *MSN2* and *MSN4* genes in corn synthesis medium using ISH as parental strains. **b** Relative transcription levels of *HSP30* gene in strains ISH::HSF1, ISH::MSN2 and ISH::MSN4. **c** Ethyl acetate concentration of strains ISH::HSF1 and ISH::HSP30::HSF1 fermented in YPD-20 medium. **d** Comparison of four mutant strains with higher ethyl acetate production than the ISP strain. The value represented mean ± SD (*n* = 3). Statistical significance is denoted as **P < 0.01, *P < 0.05. The lowercase letters a, b, c and d indicate significant differences as determined using Ducan’s multiple range test (*P* < 0.05)

As shown in Fig. 8a, the transcription factors Hsf1, Msn2 and Msn4 were differentially regulated for ethyl acetate production in corn synthesis medium. Compared with the ISH strain, ethyl acetate production was increased by 49.81% for ISH::HSF1, but the concentrations of ISH::MSN2 and ISH::MSN4 were decreased by 21.69% and 36.29%, respectively. The relative expression of *HSP30* gene in strains ISH::HSF1, ISH::MSN2 and ISH::MSN4 was 2.19-, 1.17- and 1.16-fold higher than that of control strain ISH, respectively. The relative expression of *HSP30* gene of strain ISH::HSF1 was obviously higher than that of strains ISH::MSN2 and ISH::MSN4, indicating that the transcript level of *HSP30* gene was mainly controlled by Hsf1 (shown in Fig. 8b). The combined overexpression of *HSF1* and *HSP30* was beneficial to further increase the ethyl acetate yield by 66.74% than that of strain ISH. The above results suggest that the combined overexpression of *HSF1* transcription factor and *HSP30* gene is necessary to improve the yield of the target product. However, overexpression of *HSF1* had a negative effect on the fermentation rate of mutant strains ISH::HSF1 and ISH::HSP30::HSF1 (Additional file 1: Fig. S8). This meant that while the alleviation of RHTS levels increased the yield of the target product, the fermentation rate was also decreased.

Furthermore, YPD-20 medium was also used to evaluate the ethyl acetate production of mutant strains. As shown in Fig. 8c, the ethyl acetate production of strains ISH::HSF1 and ISH::HSP30::HSF1 increased by 57.3% and 68.1%, respectively, compared with that of strain ISH. The above experimental results proved that the co-expression of *HSP30* and *HSF1* further increased the production of ethyl acetate, whether the strain was cultured in corn synthetic medium or YPD-20 medium.

Due to the deletion of *SSA1* gene also resulted in enhanced ethyl acetate production (as shown in Fig. 6a), strain ISH::HSP30ΔSSA1::HSF1 was constructed to verify whether the combination of *SSA1* deletion and *HSF1* overexpression further enhance ethyl acetate production. However, the concentration of ethyl acetate in strain ISH::HSP30ΔSSA1::HSF1 did not increase significantly compared to ISH::HSP30ΔSSA1. As shown in Fig. 8d, three parental strains (ISP, ISH, ISH::HSP30) and four strains (ISHΔSSA1, ISH::HSF1, ISH::HSP30::HSF1 and ISH::HSP30ΔSSA1::HSF1) with high yield of ethyl acetate were fermented in the same batch of corn synthetic medium. Strain ISH::HSP30::HSF1 had the highest production of ethyl acetate, which was 8.13% higher than strain ISP. This indicated that the overexpression of *HSF1* and *HSP30* alleviated the stress pressure of *S. cerevisiae* and ethyl acetate yield was significantly improved.

### *HSF1* overexpression improved the transcription level of target genes

**Fig. 9:**
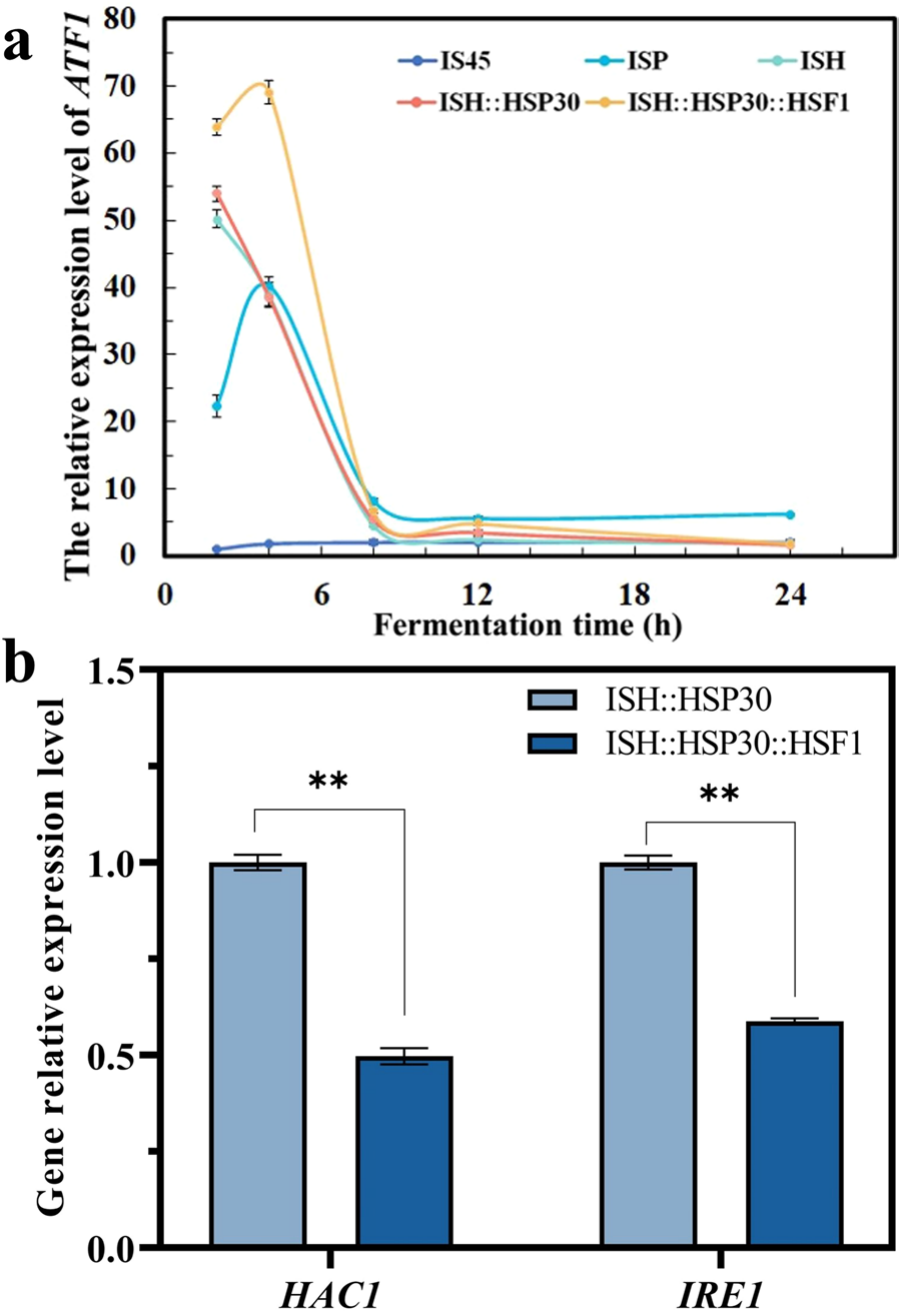
Overexpression of *HSF1* alleviated the repression of the transcription level of target gene *ATF1*. **a** *ATF1* transcription levels of strains IS45, ISP, ISH, ISH::HSP30 and ISH::HSP30::HSF1 were detected by RT-PCR at 2, 4, 8, 12, 24 h. Sample IS45-2h was used to calculate the relative transcription level of *ATF1* gene in other samples. Error bars represent standard deviation among three technical replicates. **b** Relative transcription levels of *HAC1* and *IRE1* genes in strain ISH::HSP30::HSF1 compared with that of strain ISH::HSP30. The value represented mean ± SD (*n* = 3). Statistical significance is denoted as **P < 0.01, *P < 0.05

Since the ethyl acetate production of strain ISH::HSP30::HSF1 was significantly increased, we supposed that the transcription level of its *ATF1* gene was detected and compared with strains IS45, ISP, ISH, ISH::HSP30. At 2, 4, 8, 12 and 24 h of fermentation, cell samples from 5 strains were collected, and their RNAs were extracted and reverse transcribed into cDNA. As shown in Fig. 9a, the *ATF1* gene transcript levels of strains ISP, ISH, ISH::HSP30, and ISH::HSP30::HSF1 were increased 22.31-, 50.21-, 53.94-, 63.85-fold, respectively, using IS45-2h as the control group. At the 4th hour of fermentation, the *ATF1* transcript levels of strains ISH and ISH::HSP30 decreased to 38.88-fold and 38.54-fold, which indicated that the expression of *ATF1* was inhibited and the cells were under transcriptional stress. Although the overexpression of *HSP30* gene elevated the yield of ethyl acetate, the trend of transcriptional level of *ATF1* gene remained similar compared with strain ISH. However, due to the overexpression of *HSF1*, the *ATF1* transcript level of strain ISH::HSP30::HSF1 was further increased, reaching 69.09-fold, which was consistent with the trend of ISP (up to 40.12-fold). At the 8th, 12th and 24th hours, the *ATF1* expression levels of the four strains all showed a downward trend. Strain ISH had the largest decline, 4.53-, 2.35-, 1.82-fold, followed by strain ISH::HSP30 with 5.4-, 3.45-, and 1.62-fold, respectively. Although the *ATF1* expression level of ISH::HSP30::HSF1 in the early stage of fermentation (2 h and 4 h) was significantly higher than that of ISP, its *ATF1* expression level decreased at 8, 12, and 24 h. These results demonstrated that overexpression of *HSF1* alleviated the transcriptional repression of *ATF1* in ISH strains and changed the trend of *ATF1* transcriptional levels at 2 h and 4 h, which was beneficial for the increased production of ethyl acetate.

Moreover, overexpression of the target gene *ATF1* was shown to affect the UPR levels of the strains as described above. Therefore, the UPR levels of strains ISH::HSP30 and ISH::HSP30::HSF1 were also examined to verify whether the overexpression of *HSF1* affected the UPR levels of the strains. As shown in Fig. 9b, the gene transcript levels of *HAC1* and *IRE1* of strain ISH::HSP30::HSF1 were decreased by 50.26% and 41.27%, respectively, compared to strain ISH::HSP30. This predicted that the overexpression of *HSF1* down-regulated the UPR level of the strain and alleviated the ER stress caused by the strong overexpression of the target genes.

### Combined overexpression of Hsf1 and Hsps reconstructs the regulation of target genes by RHTS

**Fig. 10:**
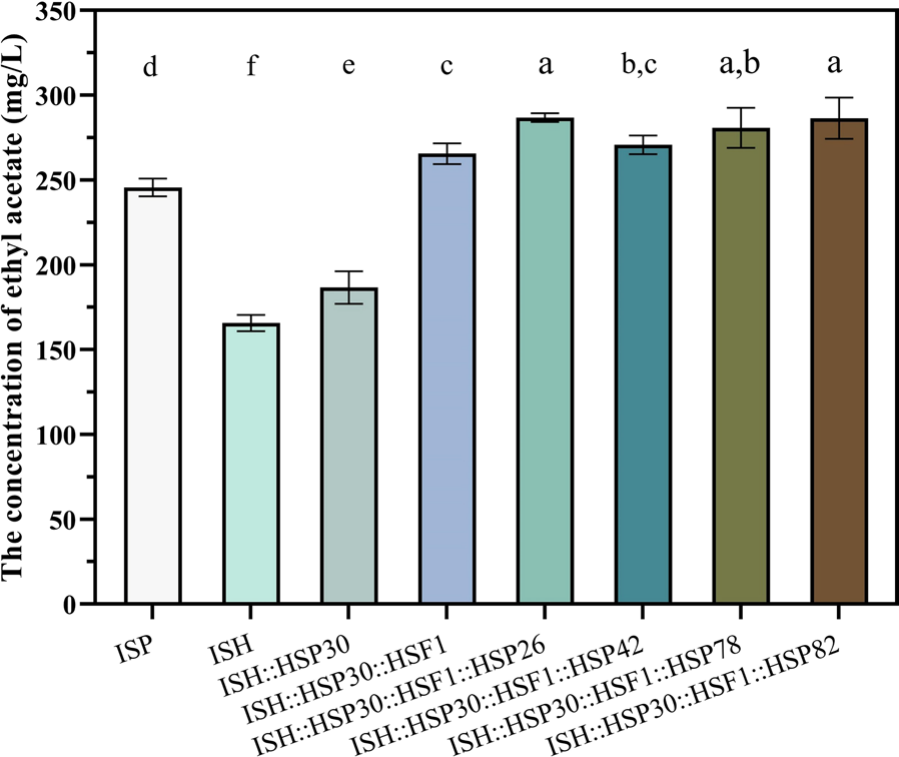
Combined overexpression of Hsf1 and Hsps reconstructs the regulation of target genes by RHTS. Effect of *HSP26*, *HSP42*, *HSP78* and *HSP82* gene overexpression on ethyl acetate production based on strain ISH::HSP30::HSF1. Error bars represent standard deviation among three technical replicates. Statistical significance is denoted as **P < 0.01, *P < 0.05

Combined overexpression of the *HSP30* and *HSF1* was shown to regulate the transcript levels of the *ATF1* gene at different timepoints and to increase the production of ethyl acetate (compared to strain ISP). In the present study, knockdown of four genes, *HSP42*, *HSP26*, *HSP78* and *HSP82*, decreased the production of ethyl acetate. Therefore, overexpression of these genes on the basis of strain ISH::HSP30::HSF1 had the potential to further increase the production of ethyl acetate. As shown in Fig. 10, the ethyl acetate yields of strains ISH::HSP30::HSF1::HSP26, ISH::HSP30::HSF1::HSP78, and ISH::HSP30::HSF1::HSP82 were 286.78, 280.75 and 286.42 mg/L, which increased by 7.97%, 5.71% and 7.83%, respectively, compared to strain ISH::HSP30::HSF1. These results indicated that further overexpression of *HSP26*, *HSP42*, *HSP78* and *HSP82* genes was beneficial to increase the production of ethyl acetate, and *HSP26* and *HSP82* had more obvious effects.

### Regulatory mechanism of kinases Rim15 and Yak1 on RHTS

In the above studies, the transcription factor Hsf1 had been shown to have a significant effect on the increase of ethyl acetate production. According the previous studies, the expression of transcription factor Hsf1 is co-regulated by kinases Yak1 and Rim15 [43]. Hsf1 is activated by Rim15 and Yak1 simultaneously when *S. cerevisiae* cells are under stress conditions, such as nutritional starvation [44, 45]. The kinases Yak1 and Rim15 overlap in function and also share the metabolic pathway TORC1 and PKA. Coincidentally, Singh et al. found that the knockout of *HSP30* gene led to the downregulation of TORC1 signal [46]. However, the regulatory mechanism of kinase Rim15 and Yak1 on RHTS remains unclear.

**Fig.11:**
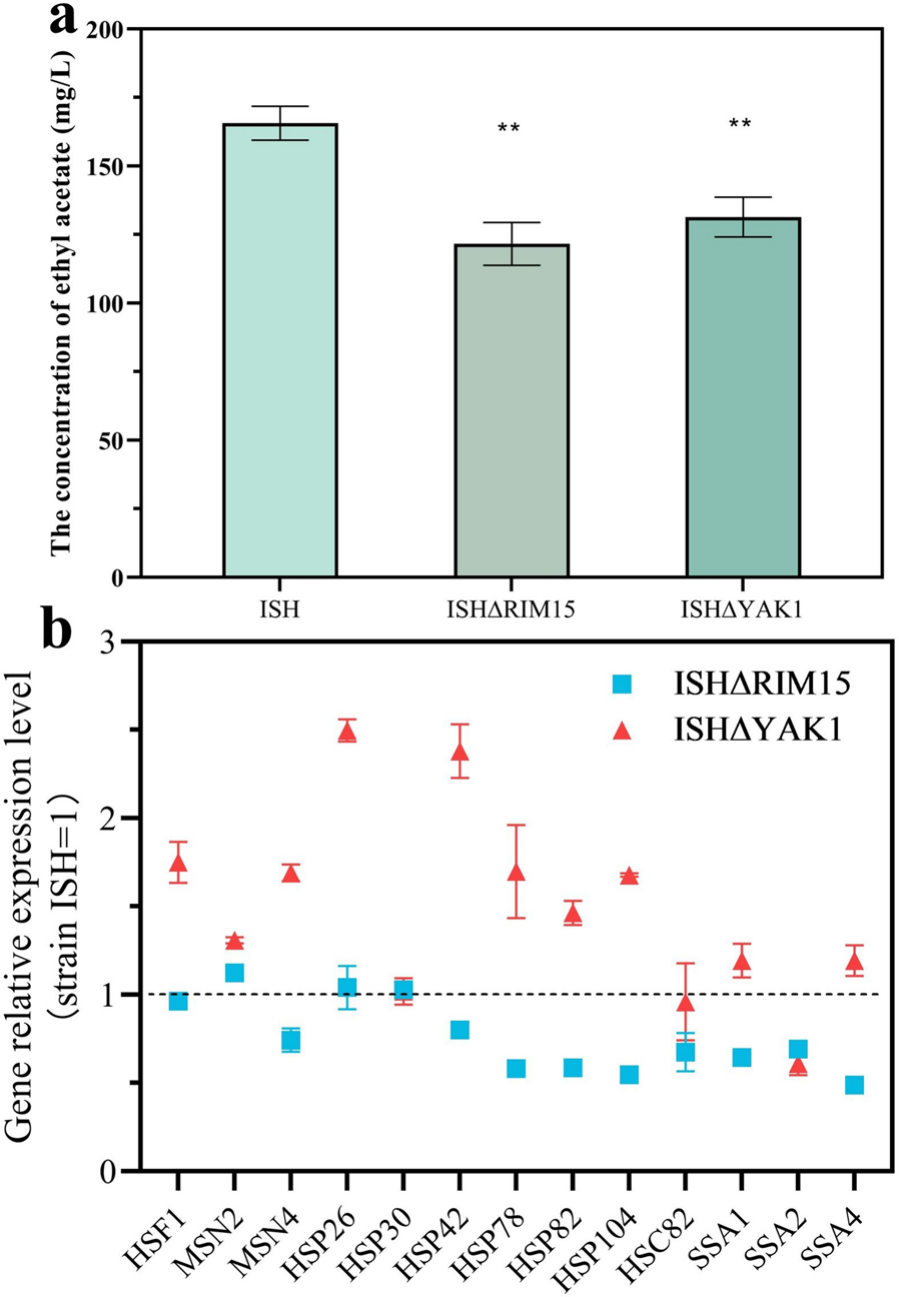
Regulation mechanism of RHTS by kinases Rim15 and Yak1. **a** Production of ethyl acetate in *RIM15* and *YAK1* gene deletion strains. **b** Relative transcript levels of key transcription factors and Hsps in strains ISH∆RIM15 and ISH∆YAK1. Transcript levels of key transcription factors and Hsps in strain ISH∆RIM15 and ISH∆YAK1, relative to strain ISH. Blue squares and red triangles represent strains ISH∆RIM15 and ISH∆YAK1, respectively. The value represented mean ± SD (*n* = 3). Statistical significance is denoted as **P < 0.01, *P < 0.05

The effect of kinase Rim15 and Yak1 on RHTS was analyzed by knockout strategy. Deletion of kinases Rim15 and Yak1 reduced the production of ethyl acetate by 20.67% and 26.56%, respectively (shown in Fig. 11a). In addition, the expression levels of transcription factors and heat shock proteins in strains ISHΔRIM15 and ISHΔYAK1 were also analyzed. As shown in Fig. 11b, the transcript levels of downstream genes were generally downregulated by *RIM15* gene deletion: the expression levels of genes *HSP78*, *HSP82*, *HSP104*, *HSC82*, *SSA1*, *SSA2* and *SSA4* decreased by 41.65%, 41.34%, 45.28%, 32.46%, 35.42%, 30.65% and 51.01%, respectively. In contrast, *YAK1* gene deletion upregulated the transcription levels of downstream genes. For example, the transcript levels of *HSP26* and *HSP42* genes were 2.49-fold and 2.38-fold higher than ISH. The differences in transcription levels of downstream genes indicated that the regulatory mechanisms of downstream genes were different between Rim15 and Yak1. The deletion of *YAK1* reduced the production of ethyl acetate, but the transcription levels of downstream genes were upregulated, suggesting that the kinase Yak1 regulated ethyl acetate production through other signaling pathways. The deletion of *RIM15* downregulated the transcription level of downstream genes, so it was speculated that Rim15 may be the key kinase regulating RHTS.

### Generality analysis of Hsf1 regulation effect on different promoter elements

**Fig. 12:**
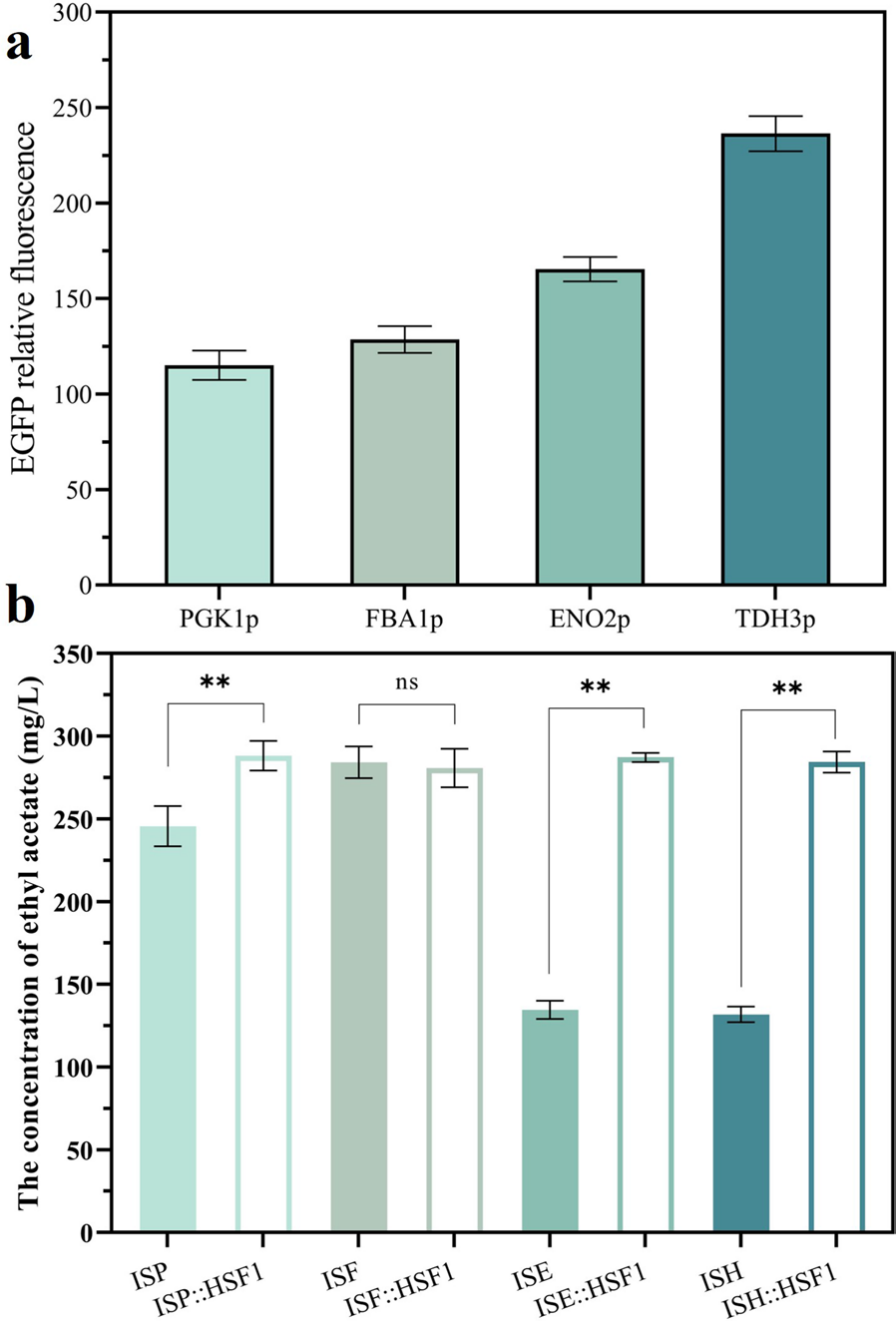
Generality analysis of Hsf1 regulation effect on different promoter elements. **a** Strength analysis of promoters *PGK1p*, *FBA1p*, *ENO2p* and *TDH3p* with EGFP as a reporter gene. **b** Regulation effect of *HSF1* overexpression on ethyl acetate production of recombinant strains constructed with different promoters. The value represented mean ± SD (*n* = 3). Statistical significance is denoted as **P < 0.01, *P < 0.05

In summary, overexpression of Hsf1 has a significant alleviating effect on RHTS induced by *TDH3p* promoter-driven target genes. Whether Hsf1 has the same regulatory effect on RHTS induced by other promoters is also a question worth exploring. Therefore, *FBA1p* and *ENO2p* were selected as target promoters and EGFP was characterized as a reporter gene for their strength. As shown in Fig. 12a, compared with *PGK1p*, the strength of *FBA1p* and *ENO2p* increased by 11.7% and 43.64%, respectively. The recombinant strains constructed by overexpressing *ATF1* gene with *FBA1p* and *ENO2p* as promoter elements were named ISF and ISE, respectively, and not only that, the recombinant strains ISF::Hsf1 and ISE::Hsf1 were constructed by overexpressing *HSF1* on the basis of strains ISF and ISE.

**Fig. 13:**
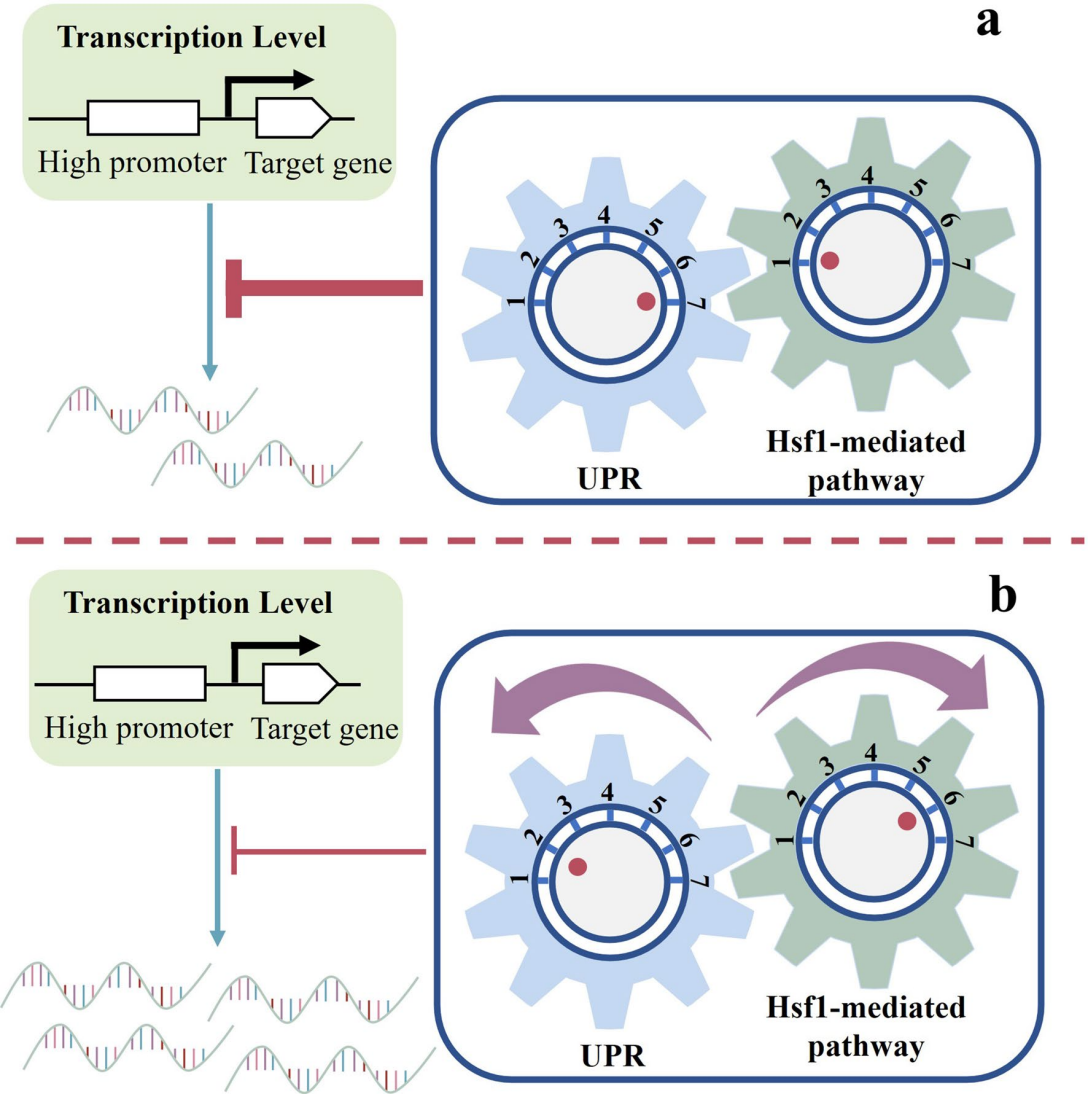
A model for regulation of target gene transcription levels by transcription factor Hsf1-mediated pathways. The down-regulation of target gene transcript levels was regulated by the linkage of the UPR pathway and the transcription factor Hsf1. When Hsf1 was not overexpressed (natural state), the UPR pathway was activated and the transcriptional levels of target genes were reduced. When Hsf1 was overexpressed, the transcriptional levels of Hsps were upregulated, the UPR pathway was repressed, and the transcriptional level of the target gene was increased

The recombinant strain ISP::HSF1, which overexpressed *HSF1* using ISP as the parental strain, was constructed and its ethyl acetate production was increased by 17.34% compared to ISP. As the strength of *ENO2p* was significantly higher than that of *PGK1p*, the ethyl acetate production of ISE was decreased by 45.18% compared to ISP, which demonstrated that overexpression of *ATF1* gene by *ENO2p* also induced RHTS. the ethyl acetate production of strain ISE::HSF1 was increased by 113.3% compared to ISE, which predicted that overexpression of *HSF1* alleviated the intracellular RHTS level of strain ISE. intracellular RHTS levels. ethyl acetate production of ISF was increased by 14.31% compared to ISP, but ethyl acetate production of ISF::HSF1 was not increased and was similar to that of ISF. This may be due to the fact that the strength of *FBA1p* was higher than *PGK1p* but weaker than *ENO2p* and *TDH3p*, and the transcript levels of *ATF1* gene driven by *FBA1p* and RHTS levels were in balance, so that ethyl acetate production was not downregulated. The strains ISP::HSF1, ISE::HSF1, and ISH::HSF1 all showed different magnitudes of elevated ethyl acetate production compared to their parental strains, demonstrating that the overexpression of Hsf1 is applicable to intracellular RHTS caused by different promoter elements and that this strategy is universal (Fig. 13).

## Discussion

Cells are exposed to unfavorable environments (e.g., unsuitable temperature and pH value) as well as nutritional limitations during growth. Correspondingly, cells have evolved elaborate and complex stress regulatory mechanisms (e.g., HSR, UPR and RESS) to resist harsh intracellular and extracellular stresses. These pathways sensitively regulate the transcriptional levels of relevant genes, efficiently maximizing the use of energy to sustain survival. In synthetic biology, regulation of the transcriptional level of the target gene is crucial, but this also trigger stressful regulatory mechanisms in the cell that are detrimental to the construction of recombinant strains or chassis microorganisms. In this study, we identified the negative effects of high transcription levels of target genes on production and analyzed the mechanism of cell response under the stress of high transcription levels using RNA-seq. Ethyl acetate is not only an important spice, but also a flavoring substance for alcoholic beverages [33, 47]. In *S. cerevisiae*, the major gene regulating ethyl acetate production is *ATF1* gene [48]. When *ATF1* was regulated by two promoters with a large difference in strength (*PGK1p* and *TDH3p*), the production of ethyl acetate by *TDH3p*-constructed strain ISH was lower than that of *PGK1p*-constructed strain ISP. The transcription level of the *ATF1* gene in strain ISH was repressed in the pre-fermentation phase compared to strain ISP. The rapid decrease in transcription levels may be the most direct reason for the lower yield. The transcription levels of *HAC1* and *IRE1* also demonstrated that the intracellular UPR level of ISH strain was significantly increased. Transcriptome sequencing analysis showed that the number of DEGs in strain ISH was higher than that in strain ISP at the three timepoints, which indicated that the metabolic flow of strain ISH was more disturbed.

Gene deletion and overexpression strategies were used to identify key genes involved in stress regulatory mechanisms. The deletion of six DEGs (*MET14*, *HOM3*, *ADR1*, *HAP4*, *BTN2*, *HSP30*) reduced the ethyl acetate production of the recombinant strain. The functions and pathways of these DEGs are different: *MET14* is involved in phosphoadenosylphosphosulphate (PAPS) synthesis, and its overexpression increased glutathione production [49, 50]; the mutant *HOM3* gene abrogated feedback inhibition, resulting in a significant increase in intracellular l-threonine concentration [51]. Transcription factors Adr1 and Hap4 have been described to be associated with cellular low pH stress and high osmotic stress, respectively [52, 53]. Therefore, we speculated that the occurrence of RHTS in strain ISH is potentially related to the pathways of these four DEGs. The deletion of DEG *HSP30* had the most obvious effect on the production of ethyl acetate. The *HSP30* gene encoding Hsp30 is a heat shock protein localized to the plasma membrane, and its transcription level is increased by ethanol induction [37]. In addition, Hsp30 regulates Pma1 H^+^-ATPase activity to prevent ATP consumption in cells under acid stress or heat shock stress [54]. Heat shock protein prevents misfolded proteins from aggregating and helps denatured proteins regain their natural conformation when *S. cerevisiae* is under stress conditions [55]. Similarly, Btn2 has been shown to play an important role in resistance to ethanol stress [56]. The promoter region of *BTN2* contains three HSEs that activate *BTN2* transcription in response to ethanol stress [57, 58]. Notably, the ethyl acetate production of strain ISH::BTN2 was also increased by 19.81% (ISH as the control group) (shown in Additional file 1: Fig. S9). This suggested that there may be a partial overlap between the functions of *BTN2* and *HSP30* in alleviating intracellular RHTS.

We also verified the alleviating effect of other Hsps on RHTS. According to the change of FPKM value, nine Hsp coding genes were verified (*HSP26*, *HSP42*, *HSP78*, *HSP82*, *HSC82*, *HSP104*, *SSA1*, *SSA2* and *SSA4*), and their expression was down-regulated in ISH strain. Deletion of the *HSP26*, *HSP42*, *HSP78*, and *HSP82* genes down-regulated ethyl acetate production, and their combined knockout with *HSP30* further reduced ethyl acetate production in the ISH strain. These results indicated that these proteins are necessary for the stress mechanism induced by strong promoters, and their complete absence or reduced expression will aggravate the stress response.

The Hsp26 and Hsp42 belong to small Hsp (sHsp), which bind to unfolded proteins and prevent their aggregation [59]. The transcriptional level of Hsp42 is 10 times higher than that of Hsp26 and is considered to be the major sHsp [55]. After pretreatment with 10% (v/v) ethanol, Hsp42 was maintained at a high level [60]. Enhanced expression of Hsp78 was helpful to deal with mitochondrial damage caused by alcohol stress, and the aggregates observed in Hsp78 knockout cells were significantly larger than those in wild-type [61]. The Hsp82 and Hsc82 are the isoforms that constitute Hsp90 and play a role in the refolding of the denatured target protein back to its natural form [55]. The transcription of *HSP82* was significantly up-regulated during high temperature fermentation (40 °C) [62]. In addition, Kim et al. found that the *HSP26*, *HSP30*, *HSP42*, *HSP82* genes were also upregulated during high-temperature fermentation of heat-resistant *S. cerevisiae* KNU5377, which contributed to the maintenance of the strain’s high heat tolerance [63]. These studies demonstrated that Hsps have different functions to maintain the normal phenotype of cells under high temperature and high concentration alcohol stress. However, according to the current report, the stressful environments to which Hsp respond include oxidative stress, pH stress, heat stress, and ethanol stress, excluding stress induced by strong promoters. To our knowledge, this study is the first to link Hsp to stress responses induced by high strength promoters.

Moreover, we tested the effect of deletion or overexpression of Hsf1, Msn2 and Msn4 on strain ISH. The study of Samakkarn et al. proved that the expression of *HSP30* gene was simultaneously regulated by Hsf1, Msn2 and Msn4 [42]. The Hsf1 is also known to be an important transcriptional regulator of heat shock proteins [64]. When cells are under stress response, the heat shock transcription factor Hsf1 activated heat shock proteins, stabilizes membranes and proteins, and inhibits protein aggregation during renaturation, effectively protecting yeast cells from stress [65]. In addition to the transcription factor Hsf1, as Msn2 and Msn4 has proven to be involved in this process [66]. Overexpression of *HSF1* gene on the basis of strain ISH successfully increased the yield of ethyl acetate (similar to that of strain ISP). It may be that the overexpression of *HSF1* activates the transcription of downstream target proteins, including heat shock proteins and unknown proteins not proven in this study. In the present study, overexpression of *MSN2* and *MSN4* did not upregulate the production of ethyl acetate. However, Richard et al. increased the recombinant protein secretion titer and product yield of *P. pastoris* by overexpressing transcription factors *MSN4* and *HAC(i)* [67]. This suggests that the variability of the chassis microorganisms, target proteins and culture conditions all lead to different effects of transcription factor regulation.

Through the combined overexpression of *HSF1*, *HSP30* and other heat shock proteins, the regulatory mechanism of RHTS on target genes was successfully reconstructed. When *HSF1* and *HSP30* were co-overexpressed, the ethyl acetate production of mutant strain ISH::HSP30::HSF1 was higher than that of strain ISP. More importantly, under the overexpression of *HSF1*, the expression level of *ATF1* gene increased at the initial stage of fermentation, which indicated that the transcriptional repression of ISH was weakened. The UPR level of strain ISH::HSP30::HSF1 was also lower than that of strain ISH::HSP30. Based on overexpression of *HSF1* and *HSP30*, further overexpression of *HSP26* and *HSP82* increased the production of ethyl acetate by 16.75% and 16.6%, respectively. Lin demonstrated that overexpression of *HAC1* alleviates the ER stress caused by overexpression of recombinant proteins, which is beneficial for protein secretion [13]. In the present study, in addition to the known response to heat stress, Hsf1 and Hsps were shown to be equally effective in relieving ER stress and improving the production of target products.

## Conclusion

In this study, we determined that the transcription factor Hsf1 and heat shock protein have alleviating effects on stress response triggered by the high strength promoter, which is manifested by a decrease in the transcription level of the target gene and a decrease in the production of the corresponding product. Mutant strains ISH::HSP30::HSF1::HSP26 and ISH::HSP30::HSF1::HSP82, with high yield of ethyl acetate were obtained by combining the strategies of overexpressing *HSF1*, *HSP30* and *HSP26*/*HSP82* genes, and their ethyl acetate production was 28.76- and 28.78-fold higher than that of wild-type strain IS45, respectively. This study reduces the negative influence caused by the high strength of promoter element and saves the material cost and time cost of screening the most suitable strength promoter. More importantly, it provides new ideas for improving the yield of high value-added products of mutant strains in metabolic engineering and synthetic biology. This will provide a convenient tool for the construction of mutant strains in metabolic engineering and synthetic biology and draw a more beautiful blueprint.

## Supplementary Information

**Additional file 1: Figure S1.** Growth curves of strains IS45, ISP and ISH. **Figure S2**. GO enrichment analysis of strains IS45, ISP and ISH at different timepoints. **Figure S3**. KEGG enrichment analysis of strains IS45, ISP and ISH at different timepoints. **Figure S4**. Gene expression validation by RT-PCR. **Figure S5**. Comparison of Fpkm values of differential genes in transcriptome sequencing. **Figure S6**. Effect of DEGs ALD2, ALD6, ADH2, ADH1 on the concentration of ethyl acetate. **Figure S7**. Growth curve of recombinant strain with Hsps family protein deleted. **Figure S8**. Fermentation rate of recombinant strains overexpressing transcription factor Hsf1. **Figure S9**. Effect of overexpression of BTN2 gene on ethyl acetate production. **Table S1**. Strains and plasmids used in this study. **Table S2**. Primers used in this study. **Table S3**. Comparison of fermentation performance of strains IS45, ISP and ISH.

## Data Availability

The data sets used and/or analyzed during the current study are available from the corresponding author on reasonable request.
