# Supplementary material for: Genome-wide analysis reveals Hsf1 maintains high transcript abundance of target genes controlled by strong constitutive promoter in Saccharomyces cerevisiae
Source: Biotechnol Biofuels Bioprod. 2023 Apr 28;16:72. doi: 10.1186/s13068-023-02322-2 (PMC10141939; doi:10.1186/s13068-023-02322-2)
Supplement: Supplementary file 1 — Additional file 1: Figure S1. Growth curves of strains IS45, ISP and ISH. Figure S2. GO enrichment analysis of strains IS45, ISP and ISH at different timepoints. Figure S3. KEGG enrichment analysis of strains IS45, ISP and ISH at different timepoints. Figure S4. Gene expression validation by RT-PCR. Figure S5. Comparison of Fpkm values of differential genes in transcriptome sequencing. Figure S6. Effect of DEGs ALD2, ALD6, ADH2, ADH1 on the concentration of ethyl acetate. Figure S7. Growth curve of recombinant strain with Hsps family protein deleted. Figure S8. Fermentation rate of recombinant strains overexpressing transcription factor Hsf1. Figure S9. Effect of overexpression of BTN2 gene on ethyl acetate production. Table S1. Strains and plasmids used in this study. Table S2. Primers used in this study. Table S3. Comparison of fermentation performance of strains IS45, ISP and ISH. [file 13068_2023_2322_MOESM1_ESM.docx]

**Fig. S1** Growth curves of strains IS45, ISP and ISH

Data represent the mean of three independent biological replicates. Error bars represent the SD of the average values.


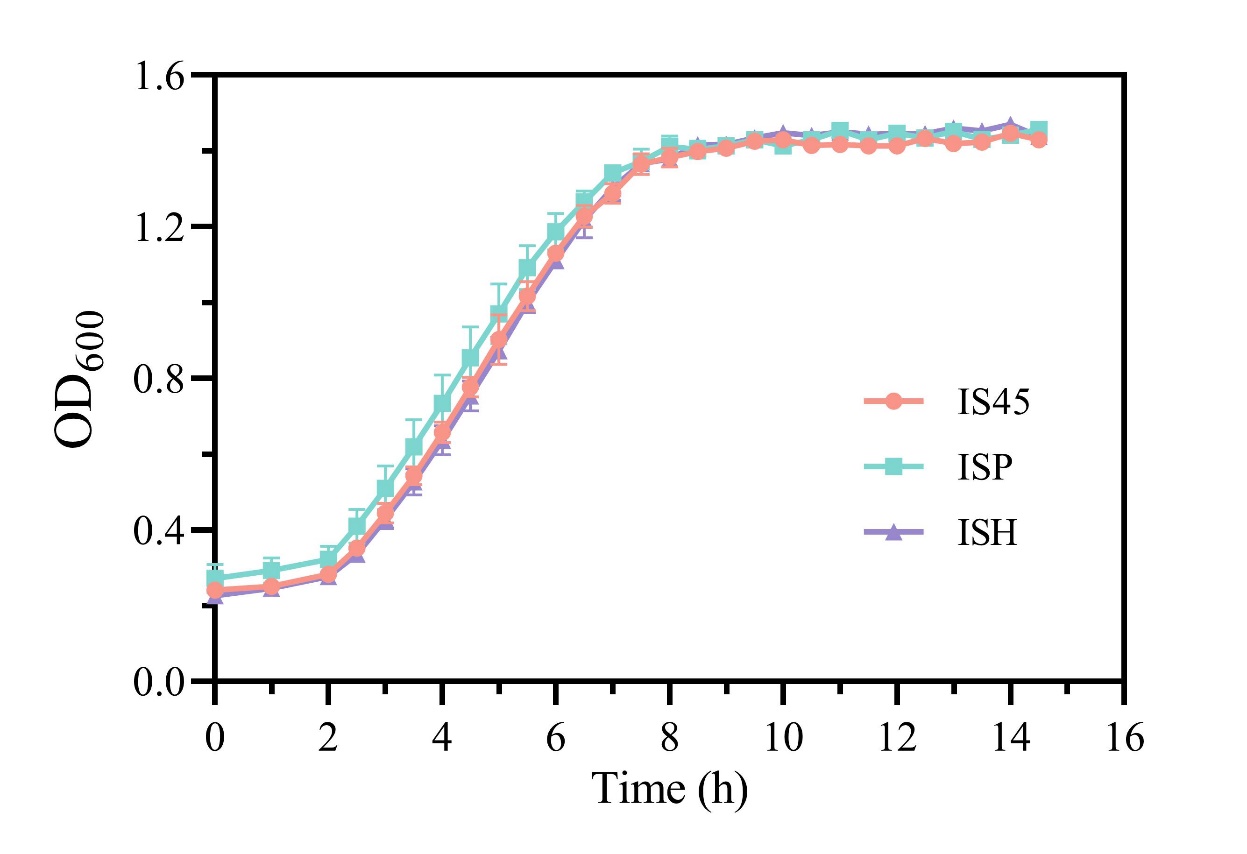


**Fig. S2** GO enrichment analysis of strains IS45, ISP and ISH at different time points

GO enrichment analysis of differentially expressed genes in mutant strains ISP and ISH compared with wild-type strain IS45 at 12 (a), 24 (b) and 36 (c) hours during fermentation


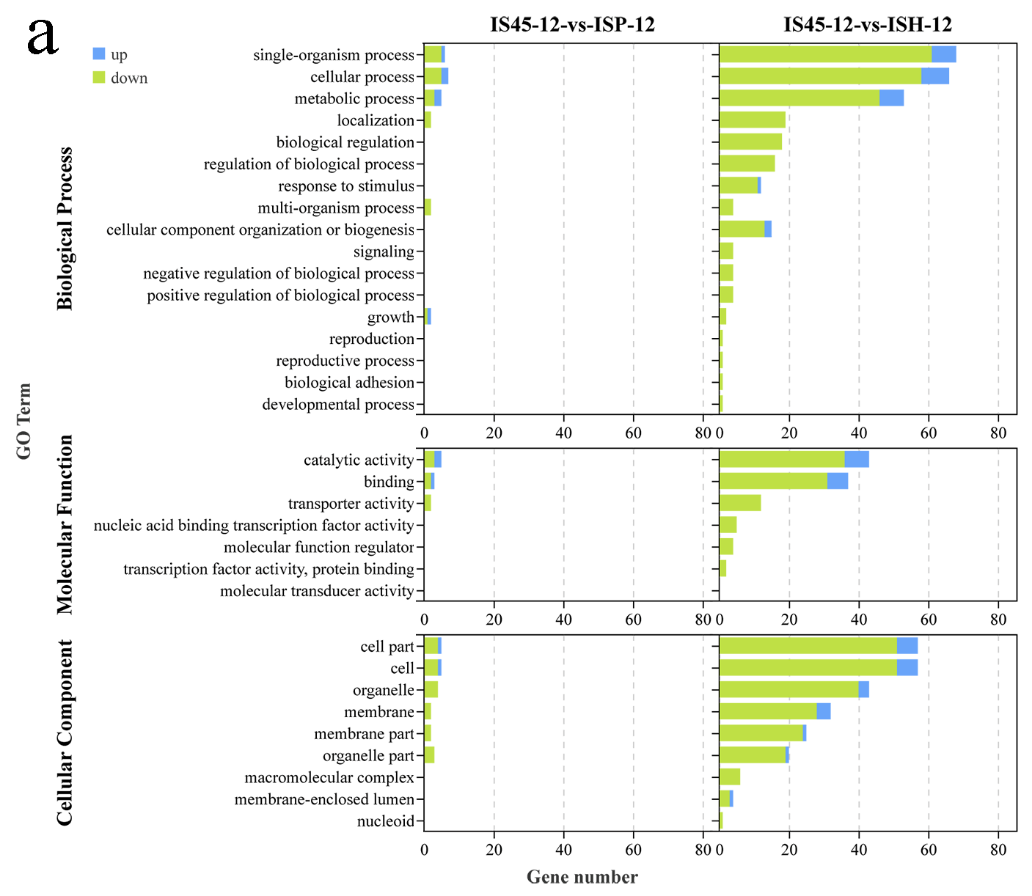


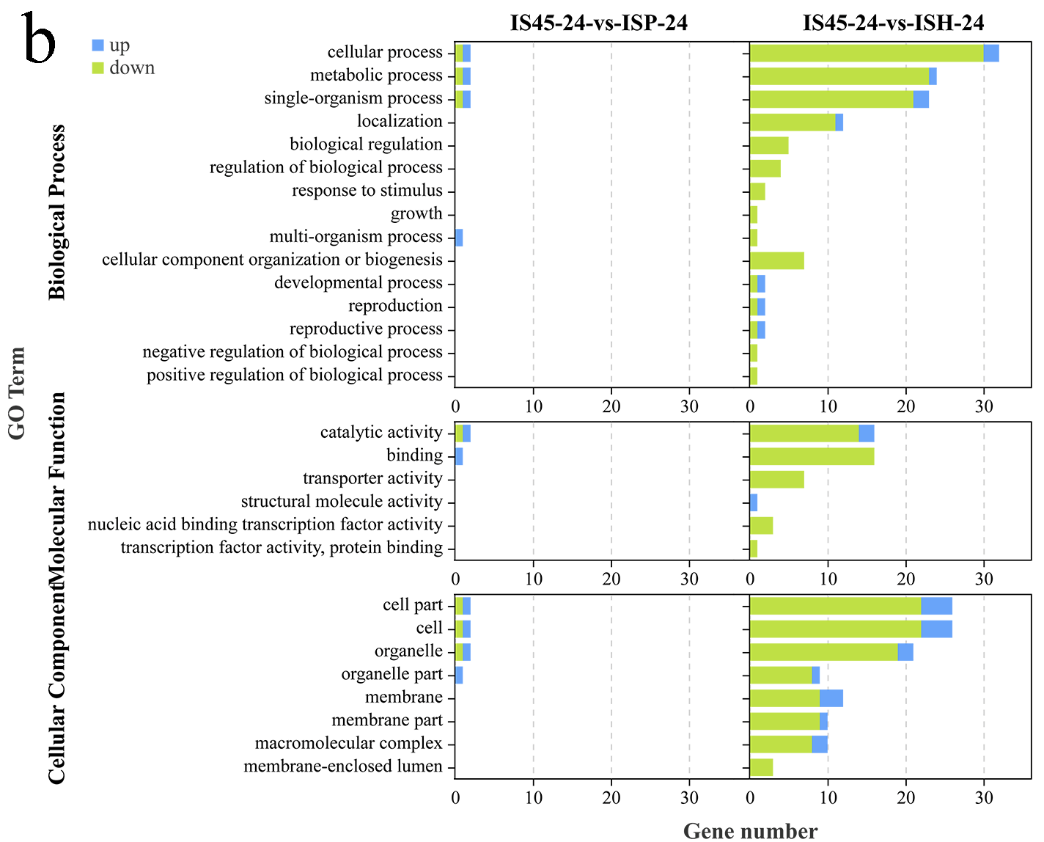


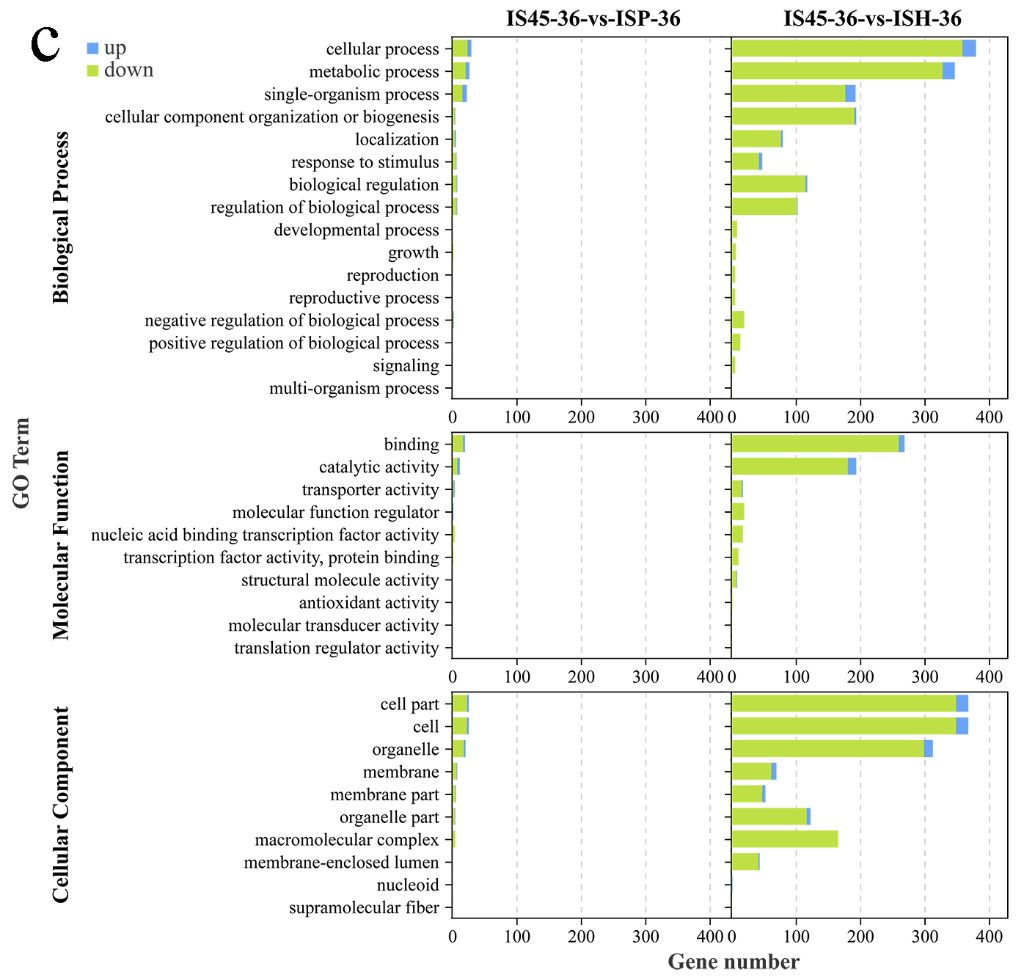


**Fig. S3** KEGG enrichment analysis of strains IS45, ISP and ISH at different time points

KEGG enrichment analysis of differentially expressed genes in mutant strains ISP and ISH compared with wild-type strain IS45 at 12 (a), 24 (b) and 36 (c) hours during fermentation


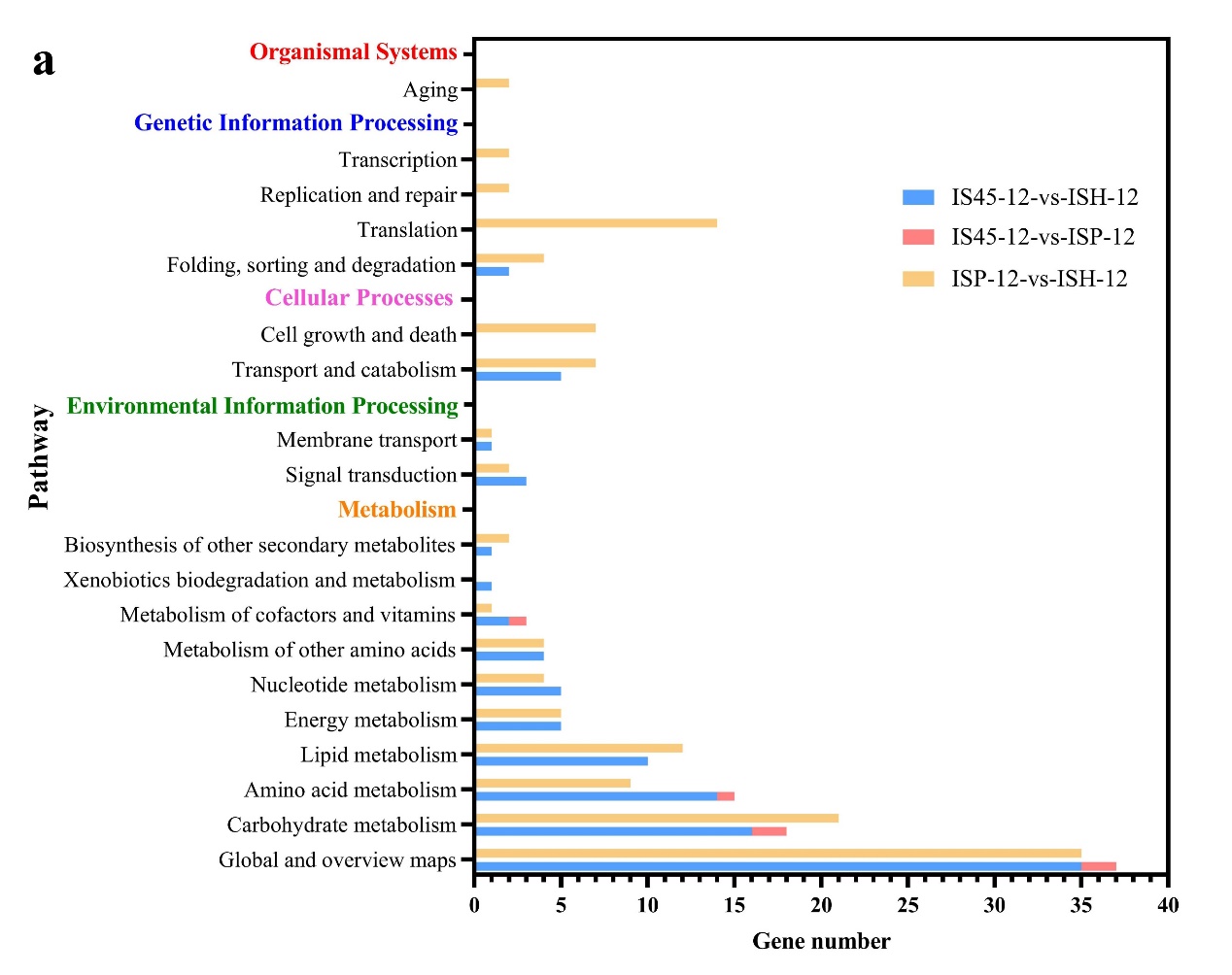

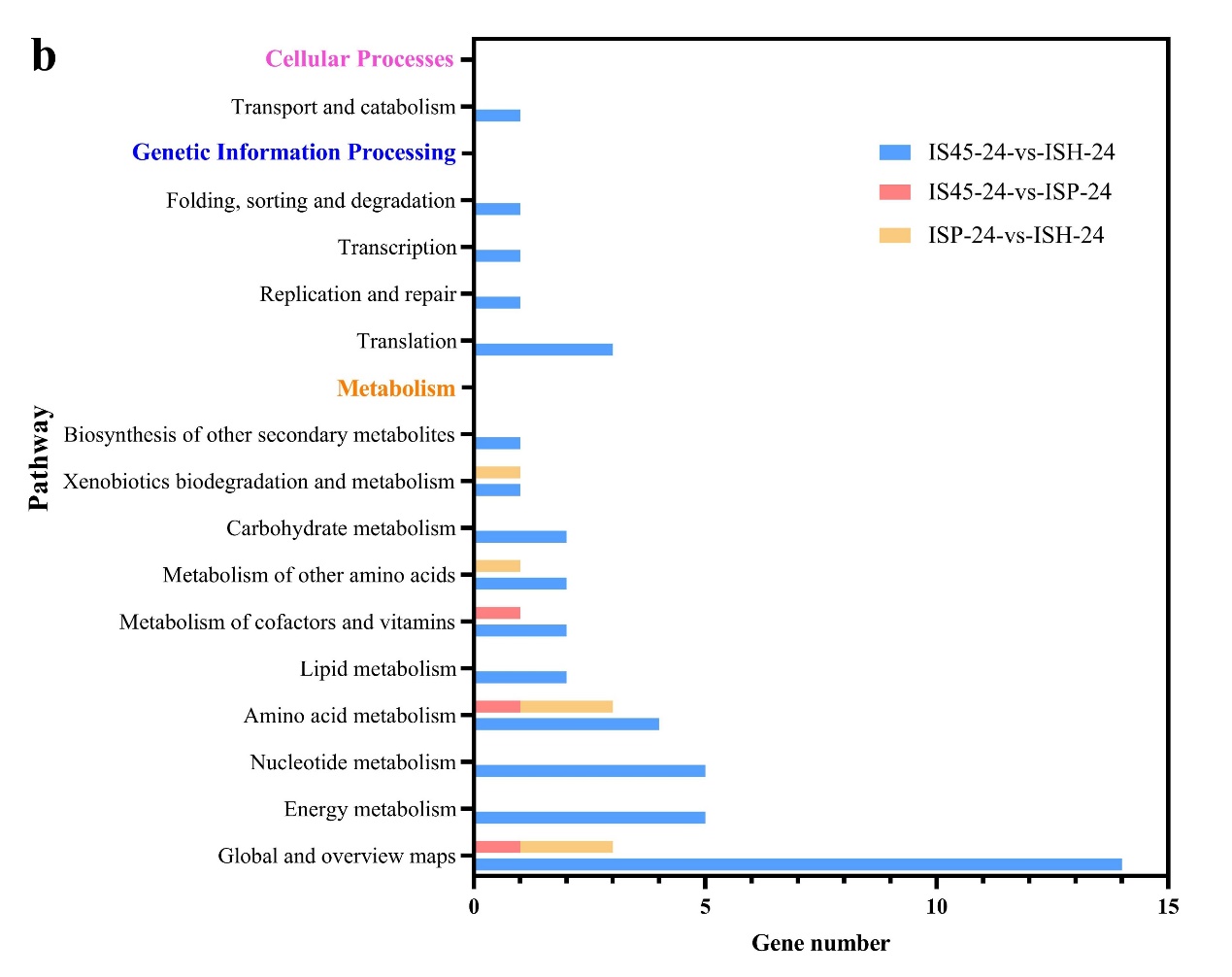

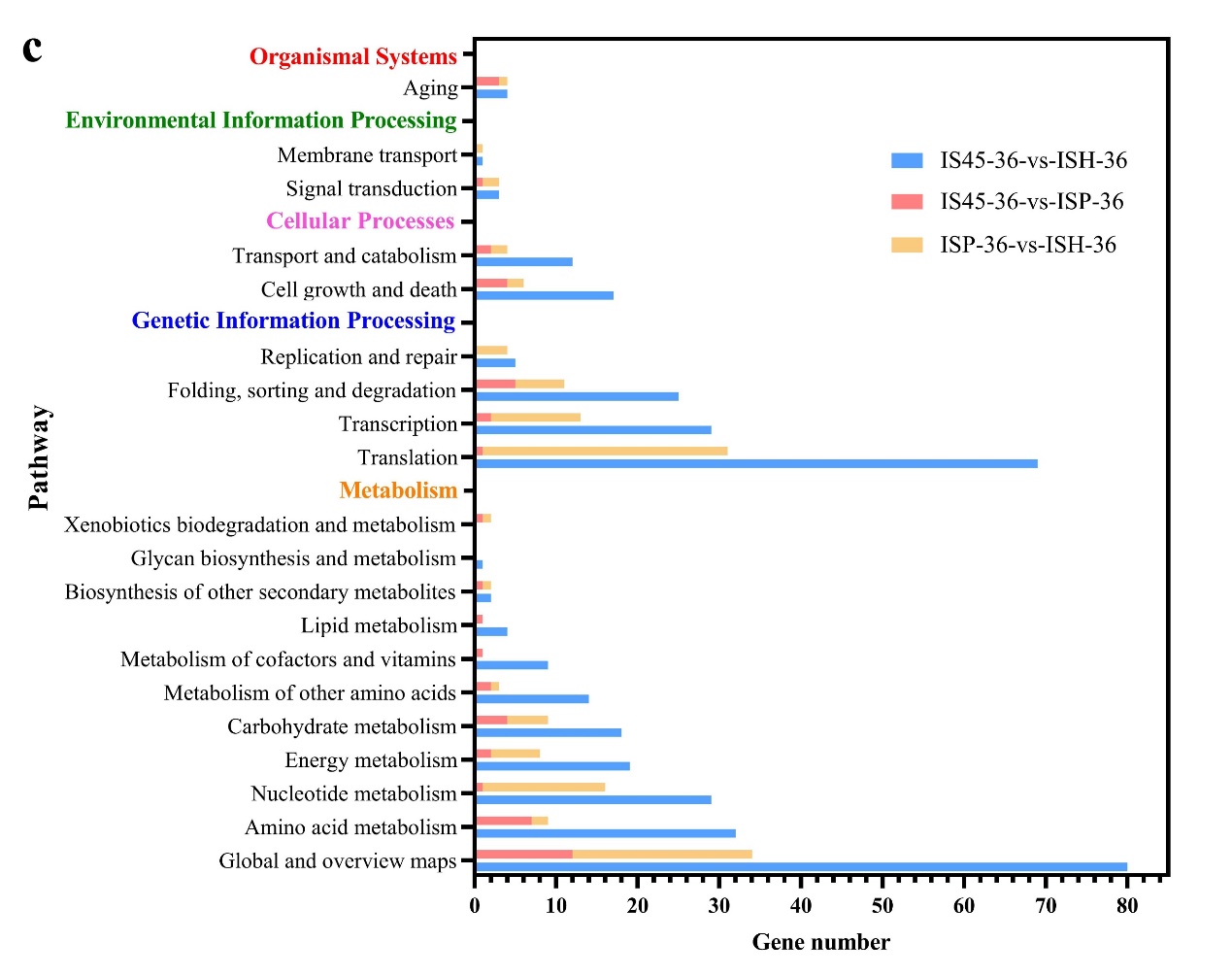


**Fig. S4** The gene expression validation by RT-PCR

Correlation of foldchange values from RNA-Seq and RT-PCR. At the 36th hour of fermentation, yeast cells from mutant strains ISP and ISH were selected as samples. The strains ISH and ISP were fermented in corn synthesis medium.


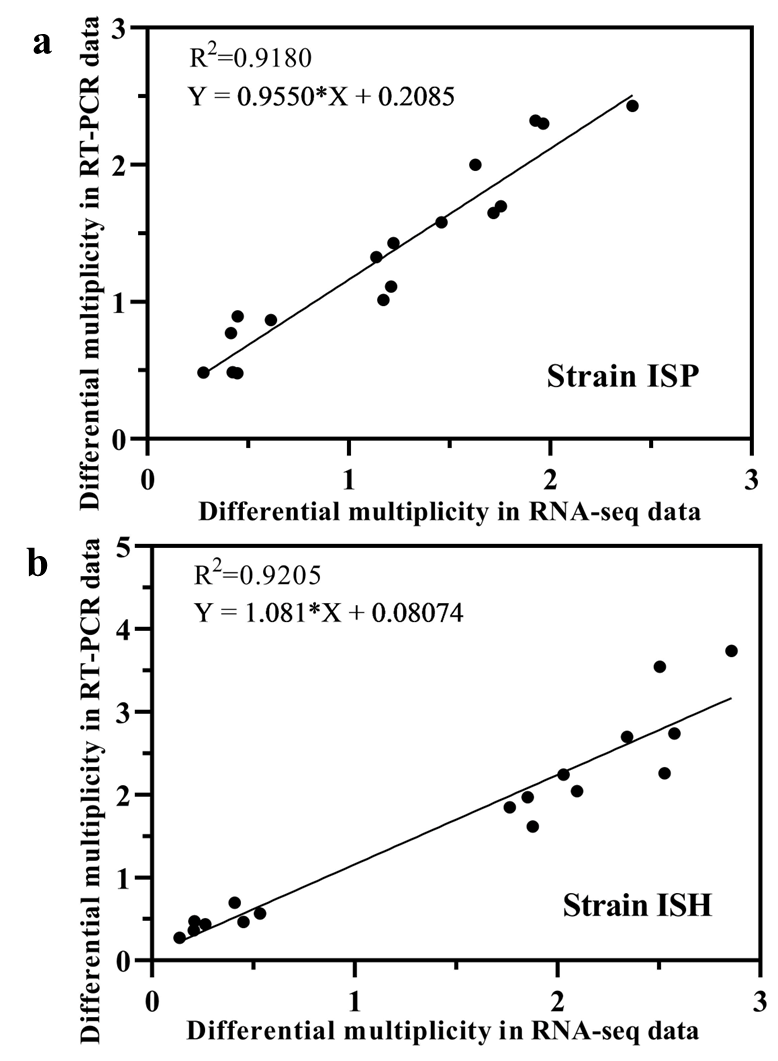


**Fig. S5** Comparison of Fpkm values of differential genes in transcriptome sequencing

The expression changes of DEGs in different strains were calculated as Log_2_(Fpkm value). The abscissa represents the time of fermentation, and the ordinate represents the DEGs of different strains. The size of the dots represents the expression level of DEGs in different strains.


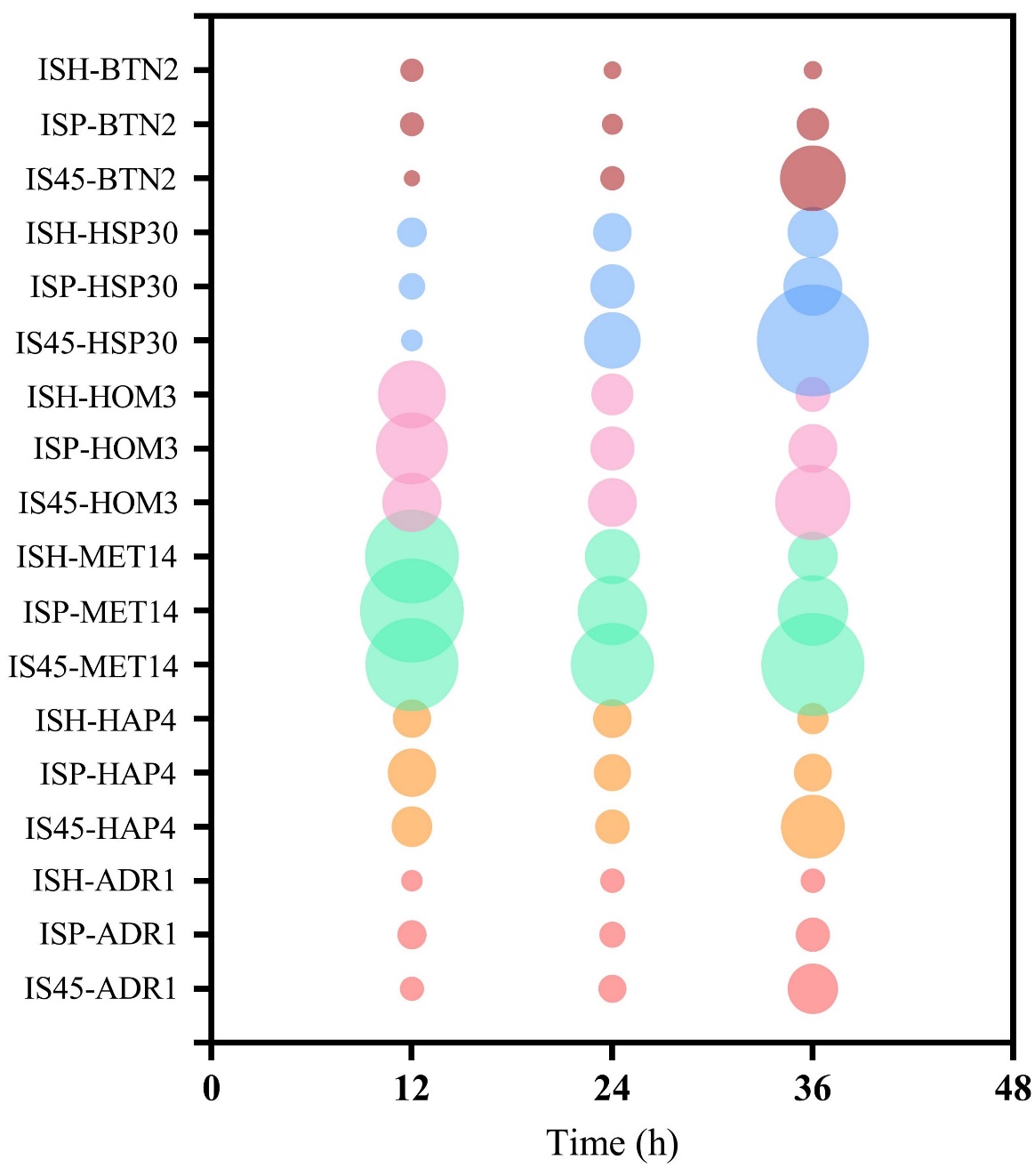


**Fig. S6** Effect of DEGs *ALD2*, *ALD6*, *ADH2*, *ADH1* on the concentration of ethyl acetate

These recombinant strains were fermented in corn synthesis medium. The value represented mean ± SD (n = 3). Statistical significance is denoted as ** = *P* <0.01, * = *P* < 0.05.

**
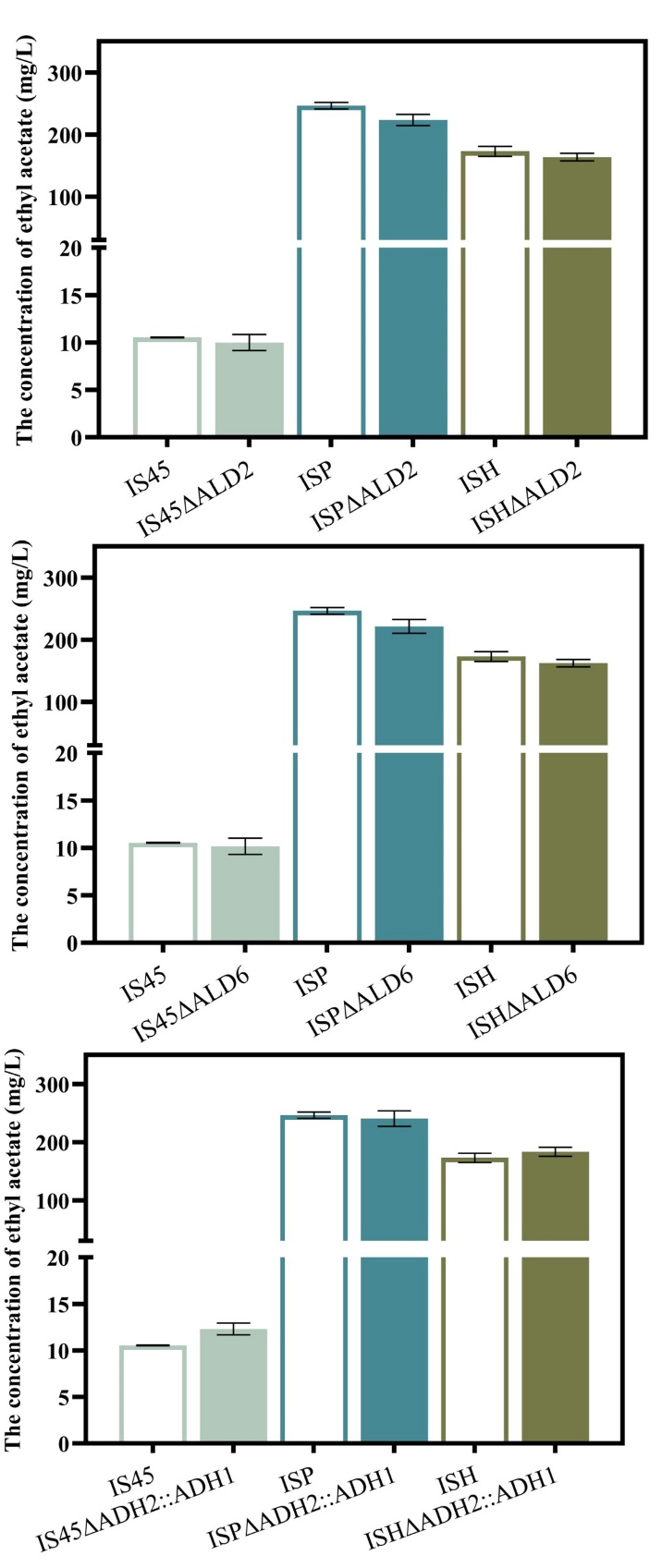
**

**Fig. S7** Growth curve of recombinant strain with Hsps family protein deleted

The abscissa represents the culture time of the strain, and the ordinate represents the OD_600_ value of the cell. Data represent the mean of three independent biological replicates. Error bars represent the SD of the average values.


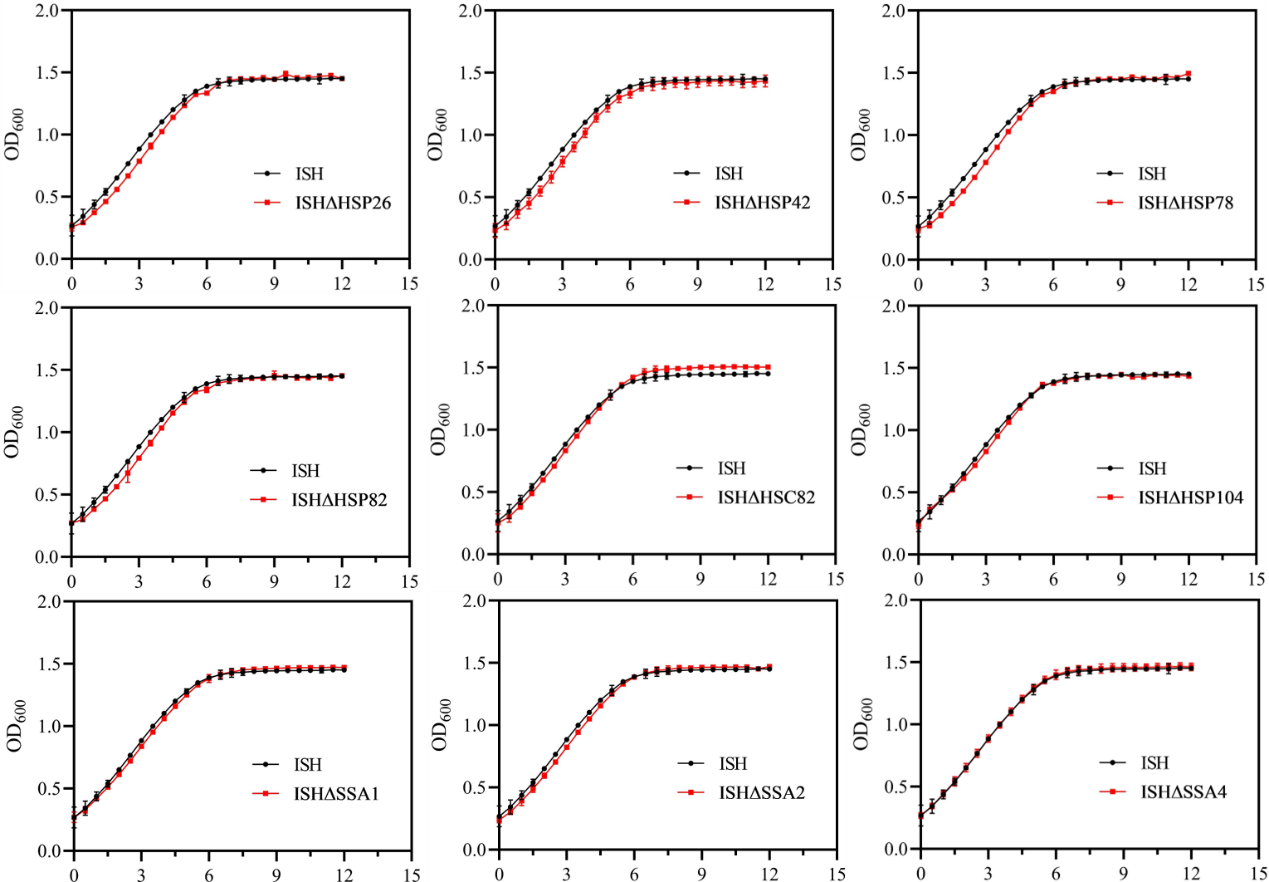


**Fig. S8** Fermentation rate of recombinant strains overexpressing transcription factor Hsf1


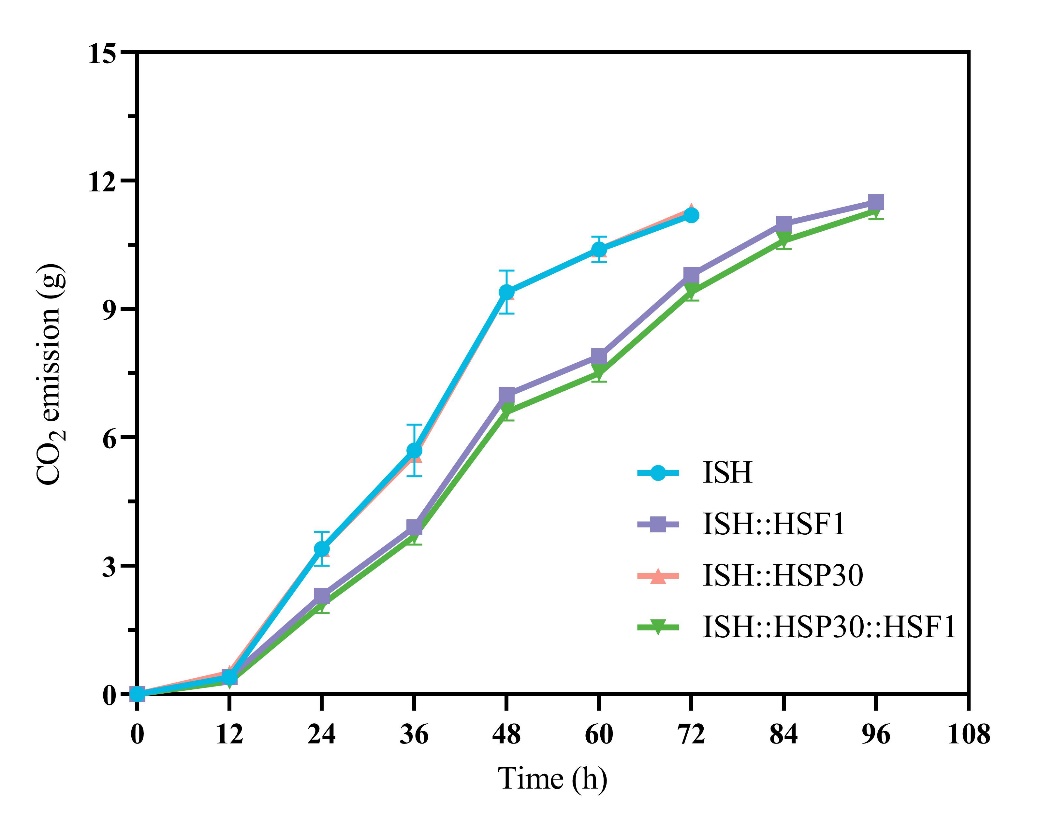


**Fig. S9** Effect of overexpression of *BTN2* gene on ethyl acetate production

The *BTN2* gene overexpression strains were fermented in corn synthesis medium with IS45, ISP, and ISH as parental strains. Error bars represent standard deviation among three technical replicates. Statistical significance is denoted as ** = *P* <0.01, * = *P* < 0.05.


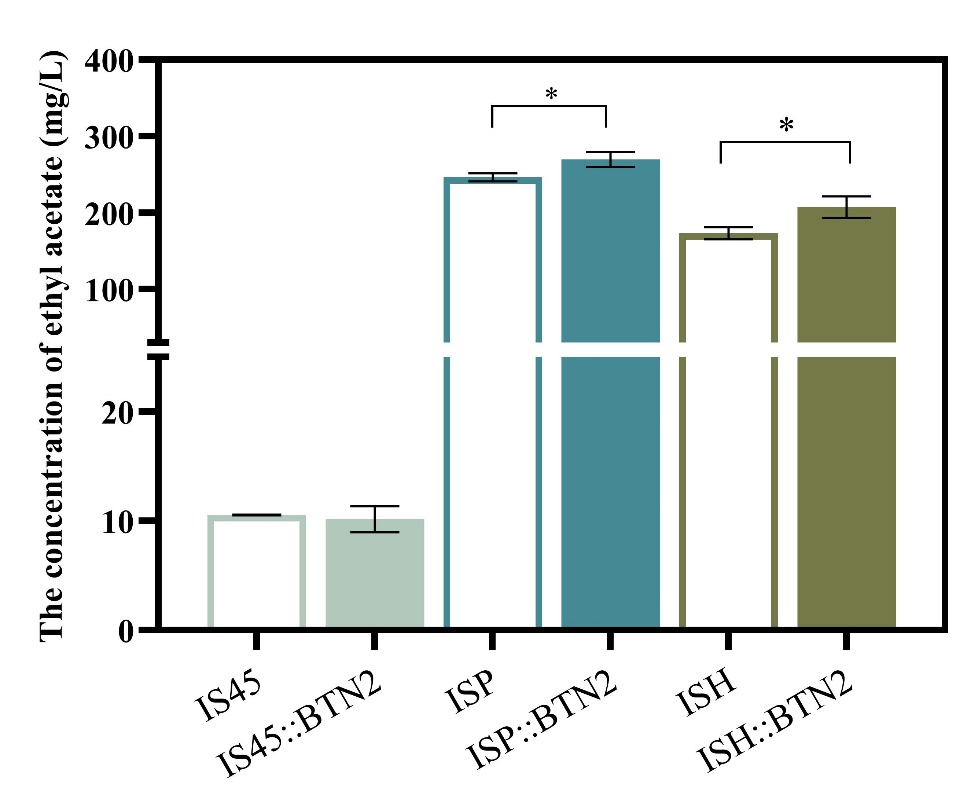


Table S1 Strains and plasmids used in this study

| Strains and plasmids | Relevant characteristics | Source |
| --- | --- | --- |
| *Saccharomyces cerevisiae* strains | | |
| IS45 | MATa, haploid yeast strain from AY12 | Angel Yeast Co.,Ltd. |
| ISP | MATa, ∆*BAT2:: PGK1p-ATF1-PGK1t-loxP* | This study |
| ISH | MATa, ∆*BAT2:: TDH3p-ATF1-PGK1t-loxP* | This study |
| ISP-RFP | IS45, ∆*BAT2:: PGK1p-RFP-PGK1t-loxP-KanMX-loxP* | This study |
| ISH-RFP | IS45, ∆*BAT2:: TDH3p-RFP-PGK1t-loxP-KanMX-loxP* | This study |
| ISP-EGFP | IS45, ∆*BAT2:: PGK1p-EGFP-PGK1t-loxP-KanMX-loxP* | This study |
| ISH-EGFP | IS45, ∆*BAT2:: TDH3p-EGFP-PGK1t-loxP-KanMX-loxP* | This study |
| ISH∆MET14 | ISH,∆*MET14::loxP-KanMX-loxP* | This study |
| ISH∆HOM3 | ISH,∆*HOM3::loxP-KanMX-loxP* | This study |
| ISH∆ADR1 | ISH,∆*ADR1::loxP-KanMX-loxP* | This study |
| ISH∆HAP4 | ISH,∆*HAP4::loxP-KanMX-loxP* | This study |
| ISH∆BTN2 | ISH,∆*BTN2::loxP-KanMX-loxP* | This study |
| ISH∆HSP30 | ISH, ∆*HSP30::loxP-KanMX-loxP* | This study |
| IS45∆HSP30 | IS45, ∆*HSP30::loxP-KanMX-loxP* | This study |
| ISP∆HSP30 | ISP, ∆*HSP30::loxP-KanMX-loxP* | This study |
| IS45::BTN2 | IS45, ∆*BTN2::PGK1p-BTN2-PGK1t-loxP-KanMX-loxP* | This study |
| IS45::HSP30 | IS45, ∆*HSP30::PGK1p-HSP30-PGK1t-loxP-KanMX-loxP* | This study |
| ISP::BTN2 | ISP, ∆*BTN2::PGK1p-BTN2-PGK1t-loxP-KanMX-loxP* | This study |
| ISP::HSP30 | ISP, ∆*HSP30::PGK1p-HSP30-PGK1t-loxP-KanMX-loxP* | This study |
| ISH::BTN2 | ISH, ∆*BTN2::PGK1p-BTN2-PGK1t-loxP-KanMX-loxP* | This study |
| ISH::HSP30 | ISH, ∆*HSP30::PGK1p-HSP30-PGK1t-loxP-KanMX-loxP* | This study |
| IS45∆ALD2 | IS45, ∆*ALD2::loxP-KanMX-loxP* | This study |
| IS45∆ALD6 | IS45, ∆*ALD6::loxP-KanMX-loxP* | This study |
| IS45∆ADH2::ADH1 | IS45, ∆*ADH2::PGK1p-ADH1-PGK1t-loxP-KanMX-loxP* | This study |
| ISP∆ALD2 | ISP, ∆*ALD2::loxP-KanMX-loxP* | This study |
| ISP∆ALD6 | ISP, ∆*ALD6::loxP-KanMX-loxP* | This study |
| ISP∆ADH2::ADH1 | ISP, ∆*ADH2::PGK1p-ADH1-PGK1t-loxP-KanMX-loxP* | This study |
| ISH∆ALD2 | ISH, ∆*ALD2::loxP-KanMX-loxP* | This study |
| ISH∆ALD6 | ISH, ∆*ALD6::loxP-KanMX-loxP* | This study |
| ISH∆ADH2::ADH1 | ISH, ∆*ADH2::PGK1p-ADH1-PGK1t-loxP-KanMX-loxP* | This study |
| ISH∆HSP26 | ISH, ∆*HSP26::loxP-KanMX-loxP* | This study |
| ISH∆HSP42 | ISH, ∆*HSP42::loxP-KanMX-loxP* | This study |
| ISH∆HSP78 | ISH, ∆*HSP78::loxP-KanMX-loxP* | This study |
| ISH∆HSP82 | ISH, ∆*HSP82::loxP-KanMX-loxP* | This study |
| ISH∆HSP104 | ISH, ∆*HSP104::loxP-KanMX-loxP* | This study |
| ISH∆HSC82 | ISH, ∆*HSC82::loxP-KanMX-loxP* | This study |
| ISH∆SSA1 | ISH, ∆*SSA1::loxP-KanMX-loxP* | This study |
| ISH∆SSA2 | ISH, ∆*SSA2::loxP-KanMX-loxP* | This study |
| ISH∆SSA4 | ISH, ∆*SSA4::loxP-KanMX-loxP* | This study |
| ISH∆HSP30∆HSP26 | ISH∆HSP30, ∆*HSP26::loxP-KanMX-loxP* | This study |
| ISH∆HSP30∆HSP42 | ISH∆HSP30, ∆*HSP42::loxP-KanMX-loxP* | This study |
| ISH∆HSP30∆HSP78 | ISH∆HSP30, ∆*HSP78::loxP-KanMX-loxP* | This study |
| ISH∆HSP30∆HSP82 | ISH∆HSP30, ∆*HSP82::loxP-KanMX-loxP* | This study |
| ISH::HSF1 | ISH, ∆*GAL80::PGK1p-HSF1-PGK1t-loxP-KanMX-loxP* | This study |
| ISH::MSN2 | ISH, ∆*GAL80::PGK1p-MSN2-PGK1t-loxP-KanMX-loxP* | This study |
| ISH::MSN4 | ISH, ∆*GAL80::PGK1p-MSN4-PGK1t-loxP-KanMX-loxP* | This study |
| ISH::HSP30::HSF1 | ISH::HSP30, ∆*GAL80::PGK1p-HSF1-PGK1t-loxP-KanMX-loxP* | This study |
| ISH::HSP30::MSN2 | ISH::HSP30, ∆*GAL80::PGK1p-MSN2-PGK1t-loxP-KanMX-loxP* | This study |
| ISH::HSP30::MSN4 | ISH::HSP30, ∆*GAL80::PGK1p-MSN4-PGK1t-loxP-KanMX-loxP* | This study |
| ISH::HSP30∆SSA1::HSF1 | ISH::HSP30, ∆*SSA1::PGK1p-HSF1-PGK1t-loxP-KanMX-loxP* | This study |
| ISH::HSP30::HSF1::HSP26 | ISH::HSP30::HSF1, ∆*HXT16::PGK1p-HSP26-PGK1t-loxP-KanMX-loxP* | This study |
| ISH::HSP30::HSF1::HSP42 | ISH::HSP30::HSF1, ∆*HXT16::PGK1p-HSP42-PGK1t-loxP-KanMX-loxP* | This study |
| ISH::HSP30::HSF1::HSP78 | ISH::HSP30::HSF1, ∆*HXT16::PGK1p-HSP78-PGK1t-loxP-KanMX-loxP* | This study |
| ISH::HSP30::HSF1::HSP82 | ISH::HSP30::HSF1, ∆*HXT16::PGK1p-HSP82-PGK1t-loxP-KanMX-loxP* | This study |
| ISH∆RIM15 | ISH, ∆*RIM15::loxP-KanMX-loxP* | This study |
| ISH∆YAK1 | ISH, ∆*YAK1::loxP-KanMX-loxP* | This study |
| ISF-EGFP | IS45, ∆*BAT2:: FBA1p-EGFP-PGK1t-loxP-KanMX-loxP* | This study |
| ISE-EGFP | IS45, ∆*BAT2:: ENO2p-EGFP-PGK1t-loxP-KanMX-loxP* | This study |
| ISF | IS45, ∆*BAT2:: FBA1p-ATF1-PGK1t-loxP-KanMX-loxP* | This study |
| ISE | IS45, ∆*BAT2:: ENO2p-ATF1-PGK1t-loxP-KanMX-loxP* | This study |
| ISF::HSF1 | ISF, ∆*GAL80::PGK1p-HSF1-PGK1t-loxP-KanMX-loxP* | This study |
| ISE::HSF1 | ISE, ∆*GAL80::PGK1p-HSF1-PGK1t-loxP-KanMX-loxP* | This study |
| plasmids | | |
| pUG6 |  | This study |
| PyEGFP3 | Ap^r^, Kan^r^, containing *EGFP -loxP-KanMX-loxP* | This study |
| pUC-PAK | Ap^r^, Kan^r^, containing *PGK1p-ATF1-PGK1t-loxP-KanMX-loxP* | This study |
| pUC57-RFP | Ap^r^, containing *RFP* reporter gene | This study |

Table S2 Primers used in this study

| Primer | Sequence (5'-3') | |  |
| --- | --- | --- | --- |
| **For the construction of recombinant strains** | |  |  |
| eTDH3-1-F | CGCTCCTTTCCAAACATCTTCGAAC | |  |
| eTDH3-1-R | ATTTACGTATTCTTTGAAATGGCAGATCGTTCTTAAAACTCGTGGAGATG | |  |
| eTDH3-2-F | CATCTCCACGAGTTTTAAGAACGATCTGCCATTTCAAAGAATACGTAAAT | |  |
| eTDH3-2-R | TGAAAAGTTCTTCTCCTTTACTCATTTTGTTTGTTTATGTGTGTTTATTC | |  |
| eTDH3-3-F | GAATAAACACACATAAACAAACAAAATGAGTAAAGGAGAAGAACTTTTCA | |  |
| eTDH3-3-R | TTTCTGAAGTCTAAGTGGGATAGGG | |  |
| eTEF1-1-F | CGCTCCTTTCCAAACATCTTCGAAC | |  |
| eTEF1-1-R | TGACGGCTGTTCCTGCCTCTGACTAATCGTTCTTAAAACTCGTGGAGATG | |  |
| eTEF1-2-F | CATCTCCACGAGTTTTAAGAACGATTAGTCAGAGGCAGGAACAGCCGTCA | |  |
| eTEF1-2-R | TGAAAAGTTCTTCTCCTTTACTCATTTTGTAATTAAAACTTAGATTAGAT | |  |
| eTEF1-3-F | ATCTAATCTAAGTTTTAATTACAAAATGAGTAAAGGAGAAGAACTTTTCA | |  |
| eTEF1-3-R | TTTCTGAAGTCTAAGTGGGATAGGG | |  |
| ePGK1-1-F | CGCTCCTTTCCAAACATCTTCGAAC | |  |
| ePGK1-1-R | TTCAGTTTTGGATAGATCAGTTAGAATCGTTCTTAAAACTCGTGGAGATG | |  |
| ePGK1-2-F | CATCTCCACGAGTTTTAAGAACGATTCTAACTGATCTATCCAAAACTGAA | |  |
| ePGK1-2-R | TGAAAAGTTCTTCTCCTTTACTCATGTTTTATATTTGTTGTAAAAAGTAG | |  |
| ePGK1-3-F | CTACTTTTTACAACAAATATAAAACATGAGTAAAGGAGAAGAACTTTTCA | |  |
| ePGK1-3-R | CCTGCAGCGTACGAAGCTTCAGCTGTAACGAACGCAGAATTTTCGAGTTA | |  |
| ePGK1-4-F | TAACTCGAAAATTCTGCGTTCGTTACAGCTGAAGCTTCGTACGCTGCAGG | |  |
| ePGK1-4-R | TACTTTACGTAGCAATAGCGATACTGCATAGGCCACTAGTGGATCTGATA | |  |
| ePGK1-5-F | TATCAGATCCACTAGTGGCCTATGCAGTATCGCTATTGCTACGTAAAGTA | |  |
| ePGK1-5-R | TTTCTGAAGTCTAAGTGGGATAGGG | |  |
| aPGK1-1-F | CGCTCCTTTCCAAACATCTTCGAAC | |  |
| aPGK1-1-R | TTCAGTTTTGGATAGATCAGTTAGAATCGTTCTTAAAACTCGTGGAGATG | |  |
| aPGK1-2-F | CATCTCCACGAGTTTTAAGAACGATTCTAACTGATCTATCCAAAACTGAA | |  |
| aPGK1-2-R | GATTTTTCTCATCGATTTCATTCATGTTTTATATTTGTTGTAAAAAGTAG | |  |
| aPGK1-3-F | CTACTTTTTACAACAAATATAAAACATGAATGAAATCGATGAGAAAAATC | |  |
| aPGK1-3-R | TTTCTGAAGTCTAAGTGGGATAGGG | |  |
| aTDH3-1-F | CGCTCCTTTCCAAACATCTTCGAAC | |  |
| aTDH3-1-R | ATTTACGTATTCTTTGAAATGGCAGATCGTTCTTAAAACTCGTGGAGATG | |  |
| aTDH3-2-F | CATCTCCACGAGTTTTAAGAACGATCTGCCATTTCAAAGAATACGTAAAT | |  |
| aTDH3-2-R | GATTTTTCTCATCGATTTCATTCATTTTGTTTGTTTATGTGTGTTTATTC | |  |
| aTDH3-3-F | GAATAAACACACATAAACAAACAAAATGAATGAAATCGATGAGAAAAATC | |  |
| aTDH3-3-R | TTTCTGAAGTCTAAGTGGGATAGGG | |  |
| aTEF1-1-F | CGCTCCTTTCCAAACATCTTCGAAC | |  |
| aTEF1-1-R | TGACGGCTGTTCCTGCCTCTGACTAATCGTTCTTAAAACTCGTGGAGATG | |  |
| aTEF1-2-F | CATCTCCACGAGTTTTAAGAACGATTAGTCAGAGGCAGGAACAGCCGTCA | |  |
| aTEF1-2-R | GATTTTTCTCATCGATTTCATTCATTTTGTAATTAAAACTTAGATTAGAT | |  |
| aTEF1-3-F | ATCTAATCTAAGTTTTAATTACAAAATGAATGAAATCGATGAGAAAAATC | |  |
| aTEF1-3-R | TTTCTGAAGTCTAAGTGGGATAGGG | |  |
| rPGK1-1-F | CGCTCCTTTCCAAACATCTTCGAAC | |  |
| rPGK1-1-R | TATCTTCTTCACCTTTTGAAACCATGTTTTATATTTGTTGTAAAAAGTAG | |  |
| rPGK1-2-F | CTACTTTTTACAACAAATATAAAACATGGTTTCAAAAGGTGAAGAAGATA | |  |
| rPGK1-2-R | AGAAAAGAAAAAAATTGATCTATCGTTATTTATATAATTCATCCATACCA | |  |
| rPGK1-3-F | TGGTATGGATGAATTATATAAATAACGATAGATCAATTTTTTTCTTTTCT | |  |
| rPGK1-3-R | TTTCTGAAGTCTAAGTGGGATAGGG | |  |
| rTDH3-1-F | CGCTCCTTTCCAAACATCTTCGAAC | |  |
| rTDH3-1-R | TATCTTCTTCACCTTTTGAAACCATTTTGTTTGTTTATGTGTGTTTATTC | |  |
| rTDH3-2-F | GAATAAACACACATAAACAAACAAAATGGTTTCAAAAGGTGAAGAAGATA | |  |
| rTDH3-2-R | TTTCTGAAGTCTAAGTGGGATAGGG | |  |
| rTEF1-1-F | CGCTCCTTTCCAAACATCTTCGAAC | |  |
| rTEF1-1-R | TATCTTCTTCACCTTTTGAAACCATTTTGTAATTAAAACTTAGATTAGAT | |  |
| rTEF1-2-F | ATCTAATCTAAGTTTTAATTACAAAATGGTTTCAAAAGGTGAAGAAGATA | |  |
| rTEF1-2-R | TTTCTGAAGTCTAAGTGGGATAGGG | |  |
| aBAT2-1-F | CGCTCCTTTCCAAACATCTTCGAAC | |  |
| aBAT2-1-R | CCTGCAGCGTACGAAGCTTCAGCTGATCGTTCTTAAAACTCGTGGAGATG | |  |
| aBAT2-2-F | CATCTCCACGAGTTTTAAGAACGATCAGCTGAAGCTTCGTACGCTGCAGG | |  |
| aBAT2-2-R | TACTTTACGTAGCAATAGCGATACTGCATAGGCCACTAGTGGATCTGATA | |  |
| aBAT2-3-F | TATCAGATCCACTAGTGGCCTATGCAGTATCGCTATTGCTACGTAAAGTA | |  |
| aBAT2-3-R | TTTCTGAAGTCTAAGTGGGATAGGG | |  |
| MET14S-F | TGTTTAGAGCAAGCGCCTTTGTGAG | |  |
| MET14S-R | CCTGCAGCGTACGAAGCTTCAGCTGTGGTGTACAGTGTGCTTGGAGAAAT | |  |
| MET14K-F | ATTTCTCCAAGCACACTGTACACCACAGCTGAAGCTTCGTACGCTGCAGG | |  |
| MET14K-R | ATTAAATGTACAGTAATCGGTCAAAGCATAGGCCACTAGTGGATCTGATA | |  |
| MET14X-F | TATCAGATCCACTAGTGGCCTATGCTTTGACCGATTACTGTACATTTAAT | |  |
| MET14X-R | ATTCATGGACGAAGCTATTGATAAC | |  |
| HOM3S-F | ATGCCCAAACTTGCCAGCCGCTCGC | |  |
| HOM3S-R | CCTGCAGCGTACGAAGCTTCAGCTGGTAAAAGTTAAAAAAAATGAAAGCT | |  |
| HOM3K-F | AGCTTTCATTTTTTTTAACTTTTACCAGCTGAAGCTTCGTACGCTGCAGG | |  |
| HOM3K-R | ATTAAAGTGAAGAAGAAAGGTGGATGCATAGGCCACTAGTGGATCTGATA | |  |
| HOM3X-F | TATCAGATCCACTAGTGGCCTATGCATCCACCTTTCTTCTTCACTTTAAT | |  |
| HOM3X-R | TTGAAAAGTTAACAAAGGTTCCTAG | |  |
| ADR1S-F | CAAAGAACAACGCCTTAAAAATAGG | |  |
| ADR1S-R | CCTGCAGCGTACGAAGCTTCAGCTGAGTAATAGAGTATGATTATTTTTTT | |  |
| ADR1K-F | AAAAAAATAATCATACTCTATTACTCAGCTGAAGCTTCGTACGCTGCAGG | |  |
| ADR1K-R | ACATATCAGCAACGAGACATAATCGGCATAGGCCACTAGTGGATCTGATA | |  |
| ADR1X-F | TATCAGATCCACTAGTGGCCTATGCCGATTATGTCTCGTTGCTGATATGT | |  |
| ADR1X-R | TAAATAAAAAAAAATTTAGAGATTC | |  |
| HAP4S-F | ATCGATTTTGCAGATTGTTCTAAAA | |  |
| HAP4S-R | CCTGCAGCGTACGAAGCTTCAGCTGGATTCTTTTTTTGTTTTTTTTTTGT | |  |
| HAP4K-F | ACAAAAAAAAAACAAAAAAAGAATCCAGCTGAAGCTTCGTACGCTGCAGG | |  |
| HAP4K-R | TTCGTTTTATTGCAACATGCCTATTGCATAGGCCACTAGTGGATCTGATA | |  |
| HAP4X-F | TATCAGATCCACTAGTGGCCTATGCAATAGGCATGTTGCAATAAAACGAA | |  |
| HAP4X-R | CCTGTCCTGTGGAGCCTGATGTAAA | |  |
| ALD2S-F | GCTACCTCTTAATGTGTCACAAGAA | |  |
| ALD2S-R | CCTGCAGCGTACGAAGCTTCAGCTGTTTTCTTTTGGCTTATTTTCACGAT | |  |
| ALD2K-F | ATCGTGAAAATAAGCCAAAAGAAAACAGCTGAAGCTTCGTACGCTGCAGG | |  |
| ALD2K-R | CACTTACATAATGATAACTATCACAGCATAGGCCACTAGTGGATCTGATA | |  |
| ALD2X-F | TATCAGATCCACTAGTGGCCTATGCTGTGATAGTTATCATTATGTAAGTG | |  |
| ALD2X-R | CAATCTCCCTGTCTCCTCCCTAAAC | |  |
| ALD6S-F | GCTCGCCTCATCCCCACGGGAATAA | |  |
| ALD6S-R | CCTGCAGCGTACGAAGCTTCAGCTGTGTATTCTGATAGTATGTGTTTGTG | |  |
| ALD6K-F | CACAAACACATACTATCAGAATACACAGCTGAAGCTTCGTACGCTGCAGG | |  |
| ALD6K-R | ACGGAAAGAAATGCAGGTTGGTACAGCATAGGCCACTAGTGGATCTGATA | |  |
| ALD6X-F | TATCAGATCCACTAGTGGCCTATGCTGTACCAACCTGCATTTCTTTCCGT | |  |
| ALD6X-R | GTTCGAAGAAGGATGTTATTATATG | |  |
| BTN2S-F | GGACTACTTTACAGGGTAATGAATA | |  |
| BTN2S-R | CCTGCAGCGTACGAAGCTTCAGCTGTTCTATATTGTAATGGGGTCTATTA | |  |
| BTN2K-F | TAATAGACCCCATTACAATATAGAACAGCTGAAGCTTCGTACGCTGCAGG | |  |
| BTN2K-R | GATGGGGAGTATGTATTATCACCCAGCATAGGCCACTAGTGGATCTGATA | |  |
| BTN2X-F | TATCAGATCCACTAGTGGCCTATGCTGGGTGATAATACATACTCCCCATC | |  |
| BTN2X-R | ACTTAATTTTTCTAAAGATGGAATT | |  |
| HSP30S-F | ATCGAAAGCGTGCTTTGTAAGAATA | |  |
| HSP30S-R | CCTGCAGCGTACGAAGCTTCAGCTGTTGAAATTTGTTGTTTTTAGTAATC | |  |
| HSP30K-F | GATTACTAAAAACAACAAATTTCAACAGCTGAAGCTTCGTACGCTGCAGG | |  |
| HSP30K-R | ATGATTAAGACAATCTCAAGCTGCTGCATAGGCCACTAGTGGATCTGATA | |  |
| HSP30X-F | TATCAGATCCACTAGTGGCCTATGCAGCAGCTTGAGATTGTCTTAATCAT | |  |
| HSP30X-R | CTTCATTACAAATGAAGAATTTCAA | |  |
| ADH2S-F | TATCTAAAAATTGCCTTATGATCCG | |  |
| ADH2S-R | TTCAGTTTTGGATAGATCAGTTAGATGTGTATTACGATATAGTTAATAGT | |  |
| A21P-F | ACTATTAACTATATCGTAATACACATCTAACTGATCTATCCAAAACTGAA | |  |
| A21P-R | CTTTTTGAGTTTCTGGGATAGACATGTTTTATATTTGTTGTAAAAAGTAG | |  |
| ADH1-F | CTACTTTTTACAACAAATATAAAACATGTCTATCCCAGAAACTCAAAAAG | |  |
| ADH1-R | AGAAAAGAAAAAAATTGATCTATCGTTATTTAGAAGTGTCAACAACGTAT | |  |
| A21TK-F | ATACGTTGTTGACACTTCTAAATAACGATAGATCAATTTTTTTCTTTTCT | |  |
| A21TK-R | AATCGTAAAGACATAAGAGATCCGCGCATAGGCCACTAGTGGATCTGATA | |  |
| ADH2X-F | TATCAGATCCACTAGTGGCCTATGCGCGGATCTCTTATGTCTTTACGATT | |  |
| ADH2X-R | TATATGAGGGTGTGTACATTGCAGT | |  |
| ::HSP30S-F | GGTGCATTGAGCCGTATTCTTCTTC | |  |
| ::HSP30S-R | TTCAGTTTTGGATAGATCAGTTAGACTAGAAAAAAACTTGTCCTTGCCAT | |  |
| ::H30P-F | ATGGCAAGGACAAGTTTTTTTCTAGTCTAACTGATCTATCCAAAACTGAA | |  |
| ::H30P-R | AAAAGCTTGATAGCGTATCGTTCATGTTTTATATTTGTTGTAAAAAGTAG | |  |
| ::HSP30-F | CTACTTTTTACAACAAATATAAAACATGAACGATACGCTATCAAGCTTTT | |  |
| ::HSP30-R | AGAAAAGAAAAAAATTGATCTATCGCTAAGCAGTATCTTCGACAGCTTGC | |  |
| ::H30TK-F | GCAAGCTGTCGAAGATACTGCTTAGCGATAGATCAATTTTTTTCTTTTCT | |  |
| ::H30TK-R | AAAAATACCATATTATCGTTTAATTGCATAGGCCACTAGTGGATCTGATA | |  |
| ::HSP30X-F | TATCAGATCCACTAGTGGCCTATGCAATTAAACGATAATATGGTATTTTT | |  |
| ::HSP30X-R | CAGTATATCGTATGTCACAGAACAA | |  |
| ::BTN2S-F | GTCGGTCAACATGCGCACGATGTTC | |  |
| ::BTN2S-R | TTCAGTTTTGGATAGATCAGTTAGAATAATGAAACATACGCTTCCCCTCC | |  |
| ::BT2P-F | GGAGGGGAAGCGTATGTTTCATTATTCTAACTGATCTATCCAAAACTGAA | |  |
| ::BT2P-R | ATGGTGAATTGAATATGGAAAACATGTTTTATATTTGTTGTAAAAAGTAG | |  |
| ::BTN2-F | CTACTTTTTACAACAAATATAAAACATGTTTTCCATATTCAATTCACCAT | |  |
| ::BTN2-R | AGAAAAGAAAAAAATTGATCTATCGTTATATCTCCTCAATAATAGAGTTT | |  |
| ::BT2TK-F | AAACTCTATTATTGAGGAGATATAACGATAGATCAATTTTTTTCTTTTCT | |  |
| ::BT2TK-R | AGTAGTAATATTAGGTATATAGAATGCATAGGCCACTAGTGGATCTGATA | |  |
| ::BTN2X-F | TATCAGATCCACTAGTGGCCTATGCATTCTATATACCTAATATTACTACT | |  |
| ::BTN2X-R | CGCTTTGGGCACGCATTCATTTGTT | |  |
| HSP26S-F | GCAGCAGCAACTCCGTGTGTACCCC | |  |
| HSP26S-R | CCTGCAGCGTACGAAGCTTCAGCTGGTTAATTTGTTTAGTTTGTTTGTTT | |  |
| HSP26K-F | AAACAAACAAACTAAACAAATTAACCAGCTGAAGCTTCGTACGCTGCAGG | |  |
| HSP26K-R | GACAACACTATAGAGCCAGGTCACTGCATAGGCCACTAGTGGATCTGATA | |  |
| HSP26X-F | TATCAGATCCACTAGTGGCCTATGCAGTGACCTGGCTCTATAGTGTTGTC | |  |
| HSP26X-R | GCTGTTTTTTACCTCCTGTAGGATC | |  |
| HSP42S-F | CTGGGGTTGGGTAACAAGTGAGCAA | |  |
| HSP42S-R | CCTGCAGCGTACGAAGCTTCAGCTGTGCTTCGGCTTGGTATGATCTTAAT | |  |
| HSP42K-F | ATTAAGATCATACCAAGCCGAAGCACAGCTGAAGCTTCGTACGCTGCAGG | |  |
| HSP42K-R | ATGTGTGTATAAACAGATACGATATGCATAGGCCACTAGTGGATCTGATA | |  |
| HSP42X-F | TATCAGATCCACTAGTGGCCTATGCATATCGTATCTGTTTATACACACAT | |  |
| HSP42X-R | AGTCTTTCAAGAGAAGATGTACCAA | |  |
| HSP78S-F | TTGAAAATACTAAAAGAAAGGAACA | |  |
| HSP78S-R | CCTGCAGCGTACGAAGCTTCAGCTGATTTAACCTTGAAAGATTTTCACTC | |  |
| HSP78K-F | GAGTGAAAATCTTTCAAGGTTAAATCAGCTGAAGCTTCGTACGCTGCAGG | |  |
| HSP78K-R | TATTATTCATAAACCGCTTGTGCAGGCATAGGCCACTAGTGGATCTGATA | |  |
| HSP78X-F | TATCAGATCCACTAGTGGCCTATGCCTGCACAAGCGGTTTATGAATAATA | |  |
| HSP78X-R | ATTTTAAACAATGTTTCATCCTTCT | |  |
| HSP82S-F | TTAAAATATAACTTAGCTTGCGTGT | |  |
| HSP82S-R | CCTGCAGCGTACGAAGCTTCAGCTGATCTTTGCGTGTTTGTTTGTGCTTT | |  |
| HSP82K-F | AAAGCACAAACAAACACGCAAAGATCAGCTGAAGCTTCGTACGCTGCAGG | |  |
| HSP82K-R | CCTATTCAAGGCCATGATGTTCTACGCATAGGCCACTAGTGGATCTGATA | |  |
| HSP82X-F | TATCAGATCCACTAGTGGCCTATGCGTAGAACATCATGGCCTTGAATAGG | |  |
| HSP82X-R | ATTAATAGATTTTAATACATTTTCT | |  |
| HSP104S-F | ACATGCGGTTGTGGCGAGAGTTTCA | |  |
| HSP104S-R | CCTGCAGCGTACGAAGCTTCAGCTGATATTCTGTATATTTTATGGTACGT | |  |
| HSP104K-F | ACGTACCATAAAATATACAGAATATCAGCTGAAGCTTCGTACGCTGCAGG | |  |
| HSP104K-R | TTTTTAAAAATCACACTATATTAAAGCATAGGCCACTAGTGGATCTGATA | |  |
| HSP104X-F | TATCAGATCCACTAGTGGCCTATGCTTTAATATAGTGTGATTTTTAAAAA | |  |
| HSP104X-R | ATTACATGAACTGCTATGGTAAAAA | |  |
| SSA1S-F | AGAAAAAAAATTTCCTTGGTTGAAA | |  |
| SSA1S-R | CCTGCAGCGTACGAAGCTTCAGCTGATTATCTGTTATTTACTTGAATTTT | |  |
| SSA1K-F | AAAATTCAAGTAAATAACAGATAATCAGCTGAAGCTTCGTACGCTGCAGG | |  |
| SSA1K-R | ATTATCAATTGCCGCACCAATTGGCGCATAGGCCACTAGTGGATCTGATA | |  |
| SSA1X-F | TATCAGATCCACTAGTGGCCTATGCGCCAATTGGTGCGGCAATTGATAAT | |  |
| SSA1X-R | TAATGATCACAGTGGATCTTTTAAA | |  |
| SSA2S-F | ACTTTTTACAAGTTCAATACCTTCT | |  |
| SSA2S-R | CCTGCAGCGTACGAAGCTTCAGCTGTGTATAAATATTTCTGTATAAAATC | |  |
| SSA2K-F | GATTTTATACAGAAATATTTATACACAGCTGAAGCTTCGTACGCTGCAGG | |  |
| SSA2K-R | ATATTTTACAGGGCGATCGCTAAGCGCATAGGCCACTAGTGGATCTGATA | |  |
| SSA2X-F | TATCAGATCCACTAGTGGCCTATGCGCTTAGCGATCGCCCTGTAAAATAT | |  |
| SSA2X-R | CAGCCATCTCCAGCGCTCTATCTGC | |  |
| SSA4S-F | ACTATTGTCACTTCTCCATTGAGAT | |  |
| SSA4S-R | CCTGCAGCGTACGAAGCTTCAGCTGGATTATTGTTTTGTTTATTTTTTTT | |  |
| SSA4K-F | AAAAAAAATAAACAAAACAATAATCCAGCTGAAGCTTCGTACGCTGCAGG | |  |
| SSA4K-R | TACTTCATCGCATCTTTGTATTTATGCATAGGCCACTAGTGGATCTGATA | |  |
| SSA4X-F | TATCAGATCCACTAGTGGCCTATGCATAAATACAAAGATGCGATGAAGTA | |  |
| SSA4X-R | TGATATTATATCTGAAACCACAGAA | |  |
| HSC82S-F | TTGAGTACAACTACGATAAGCTAAA | |  |
| HSC92S-R | CCTGCAGCGTACGAAGCTTCAGCTGATTTCAGAATGATTCTATTTTTCTA | |  |
| HSC82K-F | TAGAAAAATAGAATCATTCTGAAATCAGCTGAAGCTTCGTACGCTGCAGG | |  |
| HSC82K-R | TAAAACATGAAGGCGAAAAAAGAGAGCATAGGCCACTAGTGGATCTGATA | |  |
| HSC82X-F | TATCAGATCCACTAGTGGCCTATGCTCTCTTTTTTCGCCTTCATGTTTTA | |  |
| HSC82X-R | GGCGGAATTGGTATGCTATTCTTGC | |  |
| HXT16S-F | TAACTCCGTCTCGTATTACAGTAAA | |  |
| HXT16S-R | TTCAGTTTTGGATAGATCAGTTAGATTTTTTCCTTTAGTTTCCTTTCCTC | |  |
| ::26PGKp-F | GAGGAAAGGAAACTAAAGGAAAAAATCTAACTGATCTATCCAAAACTGAA | |  |
| ::26PGKp-R | CAAAAAATGGACTGTTAAATGACATGTTTTATATTTGTTGTAAAAAGTAG | |  |
| ::HSP26-F | CTACTTTTTACAACAAATATAAAACATGTCATTTAACAGTCCATTTTTTG | |  |
| ::HSP26-R | AGAAAAGAAAAAAATTGATCTATCGTTAGTTACCCCACGATTCTTGAGAA | |  |
| ::26PTK-F | TTCTCAAGAATCGTGGGGTAACTAACGATAGATCAATTTTTTTCTTTTCT | |  |
| ::26PTK-R | CATGTTTATCAAGCGCGCATATTGAGCATAGGCCACTAGTGGATCTGATA | |  |
| HXT16X-F | TATCAGATCCACTAGTGGCCTATGCTCAATATGCGCGCTTGATAAACATG | |  |
| HXT16X-R | GTTATCTCTTCTAATATGATGGTTG | |  |
| ::42PGKp-F | GAGGAAAGGAAACTAAAGGAAAAAATCTAACTGATCTATCCAAAACTGAA | |  |
| ::42PGKp-R | ATAGGGATGGTTGATAAAAACTCATGTTTTATATTTGTTGTAAAAAGTAG | |  |
| ::HSP42-F | CTACTTTTTACAACAAATATAAAACATGAGTTTTTATCAACCATCCCTAT | |  |
| ::HSP42-R | AGAAAAGAAAAAAATTGATCTATCGTCAATTTTCTACCGTAGGGTTGGGA | |  |
| ::42PTK-F | TCCCAACCCTACGGTAGAAAATTGACGATAGATCAATTTTTTTCTTTTCT | |  |
| ::42PTK-R | CATGTTTATCAAGCGCGCATATTGAGCATAGGCCACTAGTGGATCTGATA | |  |
| ::78PGKp-F | GAGGAAAGGAAACTAAAGGAAAAAATCTAACTGATCTATCCAAAACTGAA | |  |
| ::78PGKp-R | GTGCTTTTGTAGCTTGTCTTAACATGTTTTATATTTGTTGTAAAAAGTAG | |  |
| ::HSP78-F | CTACTTTTTACAACAAATATAAAACATGTTAAGACAAGCTACAAAAGCAC | |  |
| ::HSP78-R | AGAAAAGAAAAAAATTGATCTATCGTTACTTTTCAGCTTCCTCTTCAACA | |  |
| ::78PTK-F | TGTTGAAGAGGAAGCTGAAAAGTAACGATAGATCAATTTTTTTCTTTTCT | |  |
| ::78PTK-R | CATGTTTATCAAGCGCGCATATTGAGCATAGGCCACTAGTGGATCTGATA | |  |
| ::82PGKp-F | GAGGAAAGGAAACTAAAGGAAAAAATCTAACTGATCTATCCAAAACTGAA | |  |
| ::82PGKp-R | GAAATTCAAAAGTTTCACTAGCCATGTTTTATATTTGTTGTAAAAAGTAG | |  |
| ::HSP82-F | CTACTTTTTACAACAAATATAAAACATGGCTAGTGAAACTTTTGAATTTC | |  |
| ::HSP82-R | AGAAAAGAAAAAAATTGATCTATCGCTAATCTACCTCTTCCATTTCGGTG | |  |
| ::82PTK-F | CACCGAAATGGAAGAGGTAGATTAGCGATAGATCAATTTTTTTCTTTTCT | |  |
| ::82PTK-R | CATGTTTATCAAGCGCGCATATTGAGCATAGGCCACTAGTGGATCTGATA | |  |
| RIM15S-F | TTGCGACTCTTTTTAGCCCTACTTA | |  |
| RIM15S-R | CCTGCAGCGTACGAAGCTTCAGCTGCTGTCTTCCTCTACTGGGCTTATCT | |  |
| RIM15K-F | AGATAAGCCCAGTAGAGGAAGACAGCAGCTGAAGCTTCGTACGCTGCAGG | |  |
| RIM15K-R | TTAATTATCTTTATCTTAAAATTTAGCATAGGCCACTAGTGGATCTGATA | |  |
| RIM15X-F | TATCAGATCCACTAGTGGCCTATGCTAAATTTTAAGATAAAGATAATTAA | |  |
| RIM15X-R | GGGAGAACTATTCTTCCAGAGGATG | |  |
| YAK1S-F | TTATTAAGATGCAGCAACCCATTCA | |  |
| YAK1S-R | CCTGCAGCGTACGAAGCTTCAGCTGGATTGCTCAAATTAGCATGTGAAAA | |  |
| YAK1K-F | TTTTCACATGCTAATTTGAGCAATCCAGCTGAAGCTTCGTACGCTGCAGG | |  |
| YAK1K-R | AAGAAAAGTCAGACAAAATAACGATGCATAGGCCACTAGTGGATCTGATA | |  |
| YAK1X-F | TATCAGATCCACTAGTGGCCTATGCATCGTTATTTTGTCTGACTTTTCTT | |  |
| YAK1X-R | GGTGTGATTGAAGGTGTGGGACTAA | |  |
| ::HSF1S-F | CAGATGGAATCCCTTCCATAGAGAG | |  |
| ::HSF1S-R | TTCAGTTTTGGATAGATCAGTTAGAGACGGGAGTGGAAAGAACGGGAAAC | |  |
| ::HSF1P-F | GTTTCCCGTTCTTTCCACTCCCGTCTCTAACTGATCTATCCAAAACTGAA | |  |
| ::HSF1P-R | TCCCTGTATTTGCAGCATTATTCATGTTTTATATTTGTTGTAAAAAGTAG | |  |
| ::HSF1-F | CTACTTTTTACAACAAATATAAAACATGAATAATGCTGCAAATACAGGGA | |  |
| ::HSF1-R | AGAAAAGAAAAAAATTGATCTATCGCTATTTCTTAGCTCGTTTGGGCAGG | |  |
| ::HSF1TK-F | CCTGCCCAAACGAGCTAAGAAATAGCGATAGATCAATTTTTTTCTTTTCT | |  |
| ::HSF1TK-R | TACTTTACGTAGCAATAGCGATACTGCATAGGCCACTAGTGGATCTGATA | |  |
| ::HSF1X-F | TATCAGATCCACTAGTGGCCTATGCAGTATCGCTATTGCTACGTAAAGTA | |  |
| ::HSF1X-R | ACCTATCACCCGGTGATAACAGCAA | |  |
| ::MSN2S-F | CAGATGGAATCCCTTCCATAGAGAG | |  |
| ::MSN2S-R | TTCAGTTTTGGATAGATCAGTTAGAGACGGGAGTGGAAAGAACGGGAAAC | |  |
| ::MSN2P-F | GTTTCCCGTTCTTTCCACTCCCGTCTCTAACTGATCTATCCAAAACTGAA | |  |
| ::MSN2P-R | TATTGAAATCATGGTCGACCGTCATGTTTTATATTTGTTGTAAAAAGTAG | |  |
| ::MSN2-F | CTACTTTTTACAACAAATATAAAACATGACGGTCGACCATGATTTCAATA | |  |
| ::MSN2-R | AGAAAAGAAAAAAATTGATCTATCGTTAAATGTCTCCATGTTTTTTATGA | |  |
| ::MSN2TK-F | TCATAAAAAACATGGAGACATTTAACGATAGATCAATTTTTTTCTTTTCT | |  |
| ::MSN2TK-R | GGGCCAAGCACAGGGCAAGATGCTTGCATAGGCCACTAGTGGATCTGATA | |  |
| ::MSN2X-F | TATCAGATCCACTAGTGGCCTATGCAAGCATCTTGCCCTGTGCTTGGCCC | |  |
| ::MSN2X-R | CACCGCATTTATTAGTATTCCTGGA | |  |
| ::MSN4S-F | CAGATGGAATCCCTTCCATAGAGAG | |  |
| ::MSN4S-R | TTCAGTTTTGGATAGATCAGTTAGAGACGGGAGTGGAAAGAACGGGAAAC | |  |
| ::MSN4P-F | GTTTCCCGTTCTTTCCACTCCCGTCTCTAACTGATCTATCCAAAACTGAA | |  |
| ::MSN4P-R | TACTATTAGGTCCGAAGACTAGCATGTTTTATATTTGTTGTAAAAAGTAG | |  |
| ::MSN4-F | CTACTTTTTACAACAAATATAAAACATGCTAGTCTTCGGACCTAATAGTA | |  |
| ::MSN4-R | AGAAAAGAAAAAAATTGATCTATCGTCAAAAATCACCGTGCTTTTTGTGA | |  |
| ::MSN4TK-F | TCACAAAAAGCACGGTGATTTTTGACGATAGATCAATTTTTTTCTTTTCT | |  |
| ::MSN4TK-R | GGGCCAAGCACAGGGCAAGATGCTTGCATAGGCCACTAGTGGATCTGATA | |  |
| ::MSN4X-F | TATCAGATCCACTAGTGGCCTATGCAAGCATCTTGCCCTGTGCTTGGCCC | |  |
| ::MSN4X-R | CACCGCATTTATTAGTATTCCTGGA | |  |
| **For the verification of recombinant strains** | | | |
| TDH3-E1-U | AGAAACCTACTCCCAAGC | |  |
| TDH3-E1-D | TGTTCACCCAGACACCTA | |  |
| TDH3-E2-U | ATCAGTTCATAGGTCCATT | |  |
| TDH3-E2-D | TGTAGTTCCCGTCATCTT | |  |
| TDH3-E3-U | ATGCGTCAATCGTATGTG | |  |
| TDH3-E3-D | AGATTCATCGGCTACTTG | |  |
| PGK1-E1-U | AGAAACCTACTCCCAAGC | |  |
| PGK1-E1-D | GTATGCGATAGTTCCTCA | |  |
| PGK1-E2-U | GTGATAGACCGCCACAGA | |  |
| PGK1-E2-D | CCCAGCAGCAGTTACAAA | |  |
| PGK1-E3-U | GGAAACATTCTCGGACAC | |  |
| PGK1-E3-D | CATTACGCTCGTCATCAA | |  |
| PGK1-E4-U | GCGTTGCCAATGATGTTA | |  |
| PGK1-E4-D | TCGCCCAGTTAGCTGTTT | |  |
| PGK1-A1-U | CCCCTCAAGATACCAGCAT | |  |
| PGK1-A1-D | ACAGTGGGCATCATAGCA | |  |
| PGK1-A2-U | CCCATCCGTTATCACAAT | |  |
| PGK1-A2-D | CCGTCAGCCAGTTTAGTC | |  |
| PGK1-A3-U | ATTTCTATGTTCGGGTTC | |  |
| PGK1-A3-D | AGATTCATCGGCTACTTG | |  |
| TDH-A-1-U | AGAAACCTACTCCCAAGC | |  |
| TDH-A-1-D | TGTTCACCCAGACACCTA | |  |
| TDH-A-2-U | CTGCCTGGAGTAAATGAT | |  |
| TDH-A-2-D | AGTACGGTGGTCTAAAGTC | |  |
| TDH-A-3-U | ATGCGTCAATCGTATGTG | |  |
| TDH-A-3-D | AGATTCATCGGCTACTTG | |  |
| PGK-R-1-F | TGCTCCCTCCAACTACTC | |  |
| PGK-R-1-R | CTCTGTGGCGGTCTATCA | |  |
| PGK-R-2-F | GAGATAACGTCGATGACT | |  |
| PGK-R-2-R | TAATGGACCACCTTTAGT | |  |
| PGK-R-3-F | TGGTGGTGTTGTTACTGT | |  |
| PGK-R-3-R | TATGTTCGGATGTGATGT | |  |
| PGK-R-4-F | GTATTGATGTTGGACGAG | |  |
| PGK-R-4-R | TCTGAAGTCTAAGTGGGAT | |  |
| TDH-R-1-F | TGCTCCCTCCAACTACTC | |  |
| TDH-R-1-R | AAACTGTTCACCCAGACA | |  |
| TDH-R-2-F | CTGCCTGGAGTAAATGAT | |  |
| TDH-R-2-R | ACTGTCAAGGAGGGTATT | |  |
| TDH-R-3-F | ATGCGTCAATCGTATGTG | |  |
| TDH-R-3-R | GAAGATCGGTCGGTTTAT | |  |
| ADR1-1-F | GTGTGCTTGCGTTGTGTG | |  |
| ADR1-1-R | TCTGTGAGGGGAGCGTTT | |  |
| ADR1-2-F | CTTATTTTTGACGAGGGG | |  |
| ADR1-2-R | CGCAGGTAGAATGCTTAC | |  |
| HAP4-1-F | GCTTTGGACCACTACCCC | |  |
| HAP4-1-R | CTGATTGCCCGACATTAT | |  |
| HAP4-2-F | CATCCTATGGAACTGCCT | |  |
| HAP4-2-R | AATCGCCTTACCGTAAAC | |  |
| HOM3-1-F | TGGCTTTACTGGGTTGTG | |  |
| HOM3-1-R | TCTGTGAGGGGAGCGTTT | |  |
| HOM3-2-F | CGCAGACCGATACCAGGA | |  |
| HOM3-2-R | AGGCGGAGGAGAATGACC | |  |
| MET14-1-F | AAACTGCTTCCTCTGTAT | |  |
| MET14-1-R | GTGAGTCTTTTCCTTACC | |  |
| MET14-2-F | TATTTTTGACGAGGGGAA | |  |
| MET14-2-R | AAGGACTCAGAGGGAGCT | |  |
| BTN2-1-F | AATGGCTATCGTTCAAAGG | |  |
| BTN2-1-R | TCAAGACTGTCAAGGAGGG | |  |
| BTN2-2-F | CTAACGCCGCCATCCAGTG | |  |
| BTN2-2-R | CGCTACCGACTACGCCACA | |  |
| HSP30-1-F | CGCTGAATACGTCCTGTC | |  |
| HSP30-1-R | CAGTGGCAAATCCTAACC | |  |
| HSP30-2-F | TCGCAGACCGATACCAGG | |  |
| HSP30-2-R | CAACAATGTAACGCTGAAAA | |  |
| ALD2-1-F | GATAAATCAGCAATAAGCGGACGAA | |  |
| ALD2-1-R | AGAACCTCAGTGGCAAATCCTAACC | |  |
| ALD2-2-F | GTTGCCAATGATGTTACAGATGAGA | |  |
| ALD2-2-R | ACTAAAGGGCGTGGAAGAAAGGTAC | |  |
| ALD6-1-F | ATACCATTGTAGAAGCAACCAGCAC | |  |
| ALD6-1-R | GAAAGAAGAACCTCAGTGGCAAATC | |  |
| ALD6-2-F | TAATATCATGCGTCAATCGTATGTG | |  |
| ALD6-2-R | GCAAACTAGGGACTAAGTCATTCTT | |  |
| D2O1-1-F | TGAGGGAATAATAGAATGTC | |  |
| D2O1-1-R | GAAAGCGGTAATAAGGAT | |  |
| D2O1-2-F | GACTTCAACTCAAGACGCACAG | |  |
| D2O1-2-R | CGGAGATAGCAACCCAGT | |  |
| D2O1-3-F | CGCCAAGGCTATGGGTTA | |  |
| D2O1-3-R | TTCGGATGTGATGTGAGAACTG | |  |
| D2O1-4-F | GGTTGTATTGATGTTGGACGAG | |  |
| D2O1-4-R | CGGTGGCGGTGTTTGACT | |  |
| ::HSP30-1-F | TGAGCAGCCCTGAAGAAG | |  |
| ::HSP30-1-R | CGGATGAAAGCGGTAATA | |  |
| ::HSP30-2-F | TCGTCACACAACAAGGTC | |  |
| ::HSP30-2-R | TAATAGCGAAACAACCCA | |  |
| ::HSP30-3-F | GCTACGGACGATGTGGAA | |  |
| ::HSP30-3-R | TGGGGATGTATGGGCTAA | |  |
| ::HSP30-4-F | TTGTATTGATGTTGGACG | |  |
| ::HSP30-4-R | AGAACGAAAGGATTGTGT | |  |
| ::BTN2-1-F | TAGCCCTCTGCTCCATTT | |  |
| ::BTN2-1-R | TGCTCTCTGCCCCTCTGT | |  |
| ::BTN2-2-F | GTGTGACAACAACAGCCT | |  |
| ::BTN2-2-R | CTTCCTCCTTCAAAGATT | |  |
| ::BTN2-3-F | AAAATCAGAACAGGAAAA | |  |
| ::BTN2-3-R | AGAAGAACCTCAGTGGCA | |  |
| ::BTN2-4-F | TTGTAGTTGTTCTATTTT | |  |
| ::BTN2-4-R | CTTTATTTCCTATTCCTT | |  |
| HSP26-1-F | GACTATGGGTCTCTCTCA | |  |
| HSP26-1-R | CAAATCCTAACCTTTTAT | |  |
| HSP26-2-F | AATCGTATGTGAATGCTG | |  |
| HSP26-2-R | GTTGGTTTGAGTTGTGTG | |  |
| HSP42-1-F | GTACTGTCCAAACTCTCT | |  |
| HSP42-1-R | AACCTTTTATATTTCTCT | |  |
| HSP42-2-F | TTTGATGCTCGATGAGTT | |  |
| HSP42-2-R | GGTTACCGTTCTGGCTGT | |  |
| HSP78-1-F | AATACTAGAAGAGCTGCT | |  |
| HSP78-1-R | AAATGATTATACATGGGG | |  |
| HSP78-2-F | CATCCTATGGAACTGCCT | |  |
| HSP78-2-R | CATCCCAAAAAATTAACG | |  |
| HSP82-1-F | AATGGCTACTAAGAGGAG | |  |
| HSP82-1-R | AAATGATTATACATGGGG | |  |
| HSP82-2-F | GTCGCTATACTGCTGTCG | |  |
| HSP82-2-R | GCTGAATCACCCTTCTCC | |  |
| HSP104-1-F | GATGAGGTAGTAGAACAA | |  |
| HSP104-1-R | AGGAAGTATATGAAAGAA | |  |
| HSP104-2-F | GTATGTGAATGCTGGTCG | |  |
| HSP104-2-R | ATAAAACGGAATGAGGGA | |  |
| SSA1-1-F | ATCTTGGAGAAATCGGTG | |  |
| SSA1-1-R | AAAATGCTTGATGGTCGG | |  |
| SSA1-2-F | TGTATTGATGTTGGACGA | |  |
| SSA1-2-R | GAGGATTGAGGATTGCTT | |  |
| SSA2-1-F | TACCAAAAGTCTTCCACC | |  |
| SSA2-1-R | AGCCCATTTATACCCATA | |  |
| SSA2-2-F | CCTGTTGAACAAGTCTGG | |  |
| SSA2-2-R | TAGGTGTCTACGTCTCTG | |  |
| SSA4-1-F | TTACTTTGTGTTGTCTCT | |  |
| SSA4-1-R | AACCTTTTATATTTCTCT | |  |
| SSA4-2-F | GAGTTTTCTCCTTCATTA | |  |
| SSA4-2-R | ATTTCTTCCATCTTTTCT | |  |
| HSC82-1-F | GATGAGAAGAAGTGCCGT | |  |
| HSC82-1-R | AAATGATTATACATGGGG | |  |
| HSC82-2-F | CGGTGAGTTTTCTCCTTC | |  |
| HSC82-2-R | CCTTTAACCTCCTCGATC | |  |
| ::26-1-F | CTTTTTCTTTATTTCTTT | |  |
| ::26-1-R | CTATTATTATCCTGCTCA | |  |
| ::26-2-F | GCTTTTAGTTGTGTATTT | |  |
| ::26-2-R | ACTCGTAGTTGTTGTCAT | |  |
| ::26-3-F | TGTCCCTGTTCCCATCTG | |  |
| ::26-3-R | ATTGGCAACGCTACCTTT | |  |
| ::26-4-F | TAATCCTGATATGAATAA | |  |
| ::26-4-R | TAAAAAGATAAAGAAACT | |  |
| ::42-1-F | CTTTTTCTTTATTTCTTT | |  |
| ::42-1-R | CTATTATTATCCTGCTCA | |  |
| ::42-2-F | AATGGGGTCTCATCTTGT | |  |
| ::42-2-R | TGGTCCTATTATTCCTGG | |  |
| ::42-3-F | CATCGTTCGCTCACCTAC | |  |
| ::42-3-R | CAGTCACATCATGCCCCT | |  |
| ::42-4-F | GTTGGACGAGTCGGAATC | |  |
| ::42-4-R | CGCAAAATGAAAAGAAGA | |  |
| ::78-1-F | TTCCACCTTAAAAGACGA | |  |
| ::78-1-R | AAACAAGATGAGACCCCA | |  |
| ::78-2-F | GCTTTTAGTTGTGTATTT | |  |
| ::78-2-R | TATTTTTTGTTCTTCTTG | |  |
| ::78-3-F | TTGACCAGTGAAAAGAGA | |  |
| ::78-3-R | TGATTATACATGGGGATG | |  |
| ::78-4-F | GTTGGACGAGTCGGAATC | |  |
| ::78-4-R | CGCAAAATGAAAAGAAGA | |  |
| ::82-1-F | CTTTTTCTTTATTTCTTT | |  |
| ::82-1-R | CTATTATTATCCTGCTCA | |  |
| ::82-2-F | ATGATGCCCACTGTGATC | |  |
| ::82-2-R | GTCTTTGGCTTCTTTTCC | |  |
| ::82-3-F | GAAGAAACTGACGAAGAA | |  |
| ::82-3-R | GGATGTGATGTGAGAACT | |  |
| ::82-4-F | ATACTAACGCCGCCATCC | |  |
| ::82-4-R | ATCTCCTGTGCCTTCCCC | |  |
| RIM15-1-F | TTCCTTGGAGGCAGAGTC | |  |
| RIM15-1-R | CAGTGGCAAATCCTAACC | |  |
| RIM15-2-F | TCGCAGACCGATACCAGG | |  |
| RIM15-2-R | GCACGAAGACGAAATAAAGAAT | |  |
| YAK1-1-F | TTCGCCCTCAAACTCAAC | |  |
| YAK1-1-R | CGCTCGTCATCAAAATCA | |  |
| YAK1-2-F | CAGACCGATACCAGGATC | |  |
| YAK1-2-R | GCCGTTAGAAAGAGAGAG | |  |
| ::HSF1-1-F | TTGCCTTTGTAGGTGTGT | |  |
| ::HSF1-1-R | TTTTGTCTGTGATGCGTT | |  |
| ::HSF1-2-F | CTGTTACTCTCTCTCTTT | |  |
| ::HSF1-2-R | CTGATGTTATTATTGCTA | |  |
| ::HSF1-3-F | ATCACACACTAAACGACA | |  |
| ::HSF1-3-R | AAAGGACAATTACAAACA | |  |
| ::HSF1-4-F | AGTTTTCTCCTTCATTAC | |  |
| ::HSF1-4-R | ACACTTAGCATCTCTCTT | |  |
| ::MSN2-1-F | GCCTTTGTAGGTGTGTCG | |  |
| ::MSN2-1-R | GCAGGTGATGATTGCTCT | |  |
| ::MSN2-2-F | TGAAAAAACCCAGACACG | |  |
| ::MSN2-2-R | CACCATTAGGTAAGGACA | |  |
| ::MSN2-3-F | TCATTTCTATCACCATTT | |  |
| ::MSN2-3-R | CAGTTTAGTCTGACCATC | |  |
| ::MSN2-4-F | CTGCCTCGGTGAGTTTTC | |  |
| ::MSN2-4-R | AGCCATTCATCGTGTTGT | |  |
| ::MSN4-1-F | GACGCTTTTTACTACCTG | |  |
| ::MSN4-1-R | GTGATGATTGCTCTCTGC | |  |
| ::MSN4-2-F | CTGTTACTCTCTCTCTTT | |  |
| ::MSN4-2-R | TACTGTTGCTACTATTTT | |  |
| ::MSN4-3-F | CAGTAGCACCACAAGGCA | |  |
| ::MSN4-3-R | TACGGGCGACAGTCACAT | |  |
| ::MSN4-4-F | TTGTAGTTGTTCTATTTT | |  |
| ::MSN4-4-R | ATCTTTATTTCTGTTTTA | |  |
| **For RT-PCR** | | | |
| qUBC6-F | GGACCTGCGGATACTCCTTAC | |  |
| qUBC6-R | TAATCGTGTGTTGGGCTTGA | |  |
| qATF1-F | ATGTCTGATGGTCGGTCTT | |  |
| qATF1-R | ATTGGTAATCCTCCTCGT | |  |
| 1qHSP30-F | TGGTGTTTTGGATTTATGT | |  |
| 1qHSP30-R | GATGCTCTTGGGCTCTCTG | |  |
| qPUT4-F | TGCCATTTTCATCCCTAA | |  |
| qPUT4-R | AGCCACCATTGTCTCCAC | |  |
| qTIR1-F | CCAAGACCTCTGCCATCT | |  |
| qTIR1-R | CCAGCACCCATACCAACA | |  |
| qHPA3-F | TGAGGAAGCCTGGAACAA | |  |
| qHPA3-R | ATGCCACGACGTCAAATG | |  |
| qGRE1-F | GCGAAACTAACACTCAAG | |  |
| qGRE1-R | TGGTTCCCACTATCATCA | |  |
| qPOR1-F | AAGGCTACAATGAACTGC | |  |
| qPOR1-R | CGAAAGAGGAACCGACAC | |  |
| qMET10-F | TGTTGTTGATTGGGTGGA | |  |
| qMET10-R | ACTGGTTGCTTTGGAGAT | |  |
| qHSP42-F | TATGAAGGCACTGAACCA | |  |
| qHSP42-R | GAGCGAACGATGAAGATT | |  |
| qHSP26-F | TAAGAGGCTACGCACCAA | |  |
| qHSP26-R | GGAAACCGAAACCAGATG | |  |
| qHSP78-F | GAGACCGACCCTGTTTCC | |  |
| qHSP78-R | CTCTTTCAGCGTCCCATA | |  |
| qDIP5-F | TTACCTCCCAACCAACTT | |  |
| qDIP5-R | TAGCGACAATAACAACCA | |  |
| qALD2-F | CAAAGAAGGAGTGGGATG | |  |
| qALD2-R | TGGGGGAATGAAGTAGCC | |  |
| qYER188W-F | CTCGCACCAGAACACAGAAA | |  |
| qYER188W-R | CCATAGCGTGATAATAGCCG | |  |
| qNIS1-F | ACCCAAAAAGAAGAGGAAAA | |  |
| qNIS1-R | GCGTTAGAAGAGCAGACAGA | |  |
| qALD6-F | ACTTCACCACCTTAGAGCCA | |  |
| qALD6-R | TCAAGATACAGACGTTACCC | |  |
| qADH1-F | TGTCGGCATGGGTGAAAA | |  |
| qADH1-R | GAACCGTCGTGGGTGTAA | |  |
| qADH2-F | TCACCAAAGAGAAGGACA | |  |
| qADH2-R | AAACCAACCAAGACAACA | |  |
| 1qHAC1-F | CGCACTCGTCGTCTGATA | |  |
| 1qHAC1-R | GTTGTTGTCTACGGCAGGT | |  |
| 1qIRE1-F | TGTGCATAGCGCCTTTCC | |  |
| 1qIRE1-R | GCCCAACAATAATTCCGG | |  |

Table S3 Comparison of fermentation performance of strains IS45, ISP and ISH

|  | Ethanol  (%, v/v, 20 °C) | Weight loss of  CO_2_ (g) | Residual reducing sugars  (g/L) |
| --- | --- | --- | --- |
| Corn synthesis medium - Resting | |  |  |
| IS45 | 9.4±0.2 | 10.6±0.14 | 5.0±0.3 |
| ISP | 9.4±0.2 | 10.6±0.07 | 5.1±0.3 |
| ISH | 9.4±0.1 | 10.4±0.14 | 4.9±0.3 |
| YPD-20 medium - Resting | |  |  |
| IS45 | 6.2±0.1 | 9.4±0.1 | 7.2±0.1 |
| ISP | 6.2±0.1 | 9.5±0.2 | 7.0±0.1 |
| ISH | 6.3±0.1 | 9.4±0.1 | 7.0±0.1 |
| YPD-20 medium - Shaking | |  |  |
| IS45 | 7.0±0.2 | 11.2±0.1 | 6.6±0.2 |
| ISP | 7.1±0.1 | 11.8±0.1 | 6.2±0.1 |
| ISH | 7.0±0.2 | 11.6±0.3 | 6.1±0.1 |
